# Force-tuned avidity of spike variant-ACE2 interactions viewed on the single-molecule level

**DOI:** 10.1038/s41467-022-35641-3

**Published:** 2022-12-24

**Authors:** Rong Zhu, Daniel Canena, Mateusz Sikora, Miriam Klausberger, Hannah Seferovic, Ahmad Reza Mehdipour, Lisa Hain, Elisabeth Laurent, Vanessa Monteil, Gerald Wirnsberger, Ralph Wieneke, Robert Tampé, Nikolaus F. Kienzl, Lukas Mach, Ali Mirazimi, Yoo Jin Oh, Josef M. Penninger, Gerhard Hummer, Peter Hinterdorfer

**Affiliations:** 1 Department of Experimental Applied Biophysics, Johannes Kepler University Linz, Linz, Austria; 2 Department of Theoretical Biophysics, Max Planck Institute of Biophysics, Frankfurt am Main, Germany; 3 Faculty of Physics, University of Vienna, Vienna, Austria; 4 Malopolska Centre of Biotechnology, Gronostajowa 7A, 30-387 Kraków, Poland; 5 Department of Biotechnology, Institute of Molecular Biotechnology, University of Natural Resources and Life Sciences, Vienna, Vienna, Austria; 6 Center for Molecular Modeling, University of Ghent, Ghent, Belgium; 7 Core Facility Biomolecular & Cellular Analysis, University of Natural Resources and Life Sciences, Vienna, Vienna, Austria; 8 Department of Laboratory Medicine, Unit of Clinical Microbiology, Karolinska Institute and Karolinska University Hospital, Stockholm, Sweden; 9 Apeiron Biologics, Vienna, Austria; 10 Institute of Biochemistry, Biocenter, Goethe University Frankfurt, Frankfurt, Germany; 11 Department of Applied Genetics and Cell Biology, Institute of Plant Biotechnology and Cell Biology, University of Natural Resources and Life Sciences, Vienna, Vienna, Austria; 12 National Veterinary Institute, Uppsala, Sweden; 13 Institute of Molecular Biotechnology of the Austrian Academy of Sciences (IMBA), Vienna, Austria; 14 Department of Medical Genetics, Life Sciences Institute, University of British Columbia, Vancouver, BC Canada; 15 Institute of Biophysics, Goethe University Frankfurt, Frankfurt am Main, Germany

**Keywords:** Single-molecule biophysics, Atomic force microscopy, Computational biophysics

## Abstract

Recent waves of COVID-19 correlate with the emergence of the Delta and the Omicron variant. We report that the Spike trimer acts as a highly dynamic molecular caliper, thereby forming up to three tight bonds through its RBDs with ACE2 expressed on the cell surface. The Spike of both Delta and Omicron (B.1.1.529) Variant enhance and markedly prolong viral attachment to the host cell receptor ACE2, as opposed to the early Wuhan-1 isolate. Delta Spike shows rapid binding of all three Spike RBDs to three different ACE2 molecules with considerably increased bond lifetime when compared to the reference strain, thereby significantly amplifying avidity. Intriguingly, Omicron (B.1.1.529) Spike displays less multivalent bindings to ACE2 molecules, yet with a ten time longer bond lifetime than Delta. Delta and Omicron (B.1.1.529) Spike variants enhance and prolong viral attachment to the host, which likely not only increases the rate of viral uptake, but also enhances the resistance of the variants against host-cell detachment by shear forces such as airflow, mucus or blood flow. We uncover distinct binding mechanisms and strategies at single-molecule resolution, employed by circulating SARS-CoV-2 variants to enhance infectivity and viral transmission.

## Abstract (web summary)

Combining high-speed AFM, single molecule recognition force spectroscopy, and molecular dynamics simulations Zhu, Canena, Sikora et al. characterize the interaction dynamics of the trimeric spike protein of SARS-CoV-2 wt, and delta and omicron variants with its entry receptor ACE2. While delta variant increases avidity by multivalent binding to ACE2, omicron variant shows an extended binding lifetime.

## Introduction

While alternative or auxiliary receptors have been suggested for SARS-CoV-2, genetic deletion experiments in mice and human cells and organoids have proven that ACE2 is key in the interaction with the SARS-CoV-2 Spike protein and hence viral infection^1–3^. Successful binding to ACE2 requires at least one of the three receptor-binding domains (RBDs) on a Spike protomer to be in an open (“up”) state. Studies on the conformational changes of Spike in the early stages of viral attachment suggest that the geometry of ACE2-bound RBD leads to the emergence of two-open-RBDs and three-RBD-bound conformations^4,5^. Moreover, the RBDs were suggested to undergo a pH-dependent switch of their orientation to adopt an all-down conformation at low pH, providing a basis for immune evasion^6^. Conversely, Spike protein engineering enables the generation of mutant proteins with RBDs locked in distinct conformations^7^.

Detailed native structures of the Spike^8^ in pre- and post-fusion conformations on the intact virus were determined using cryogenic electron tomography (cryo-ET)^9^. In combination with molecular dynamics simulations (MDS), this technique also revealed that the stalk domain of Spike contains three hinges, giving the head unexpected orientational freedom for scanning the cell surface^10^ to enhance receptor binding. Yet, the time-resolved motional flexibility of the Spike protein in near-physiological conditions remains to be investigated and little is known about how the RBDs can switch their geometry and orientation dynamically. Although the interaction of soluble RBD and the S1 subunit of the Spike with ACE2 has been investigated by force measurements^11–13^, biolayer interferometry^14^, and surface plasmon resonance (SPR)^15^, the dynamics of bond formation between three RBDs of a Spike protomer with ACE2, as well as the assessment of kinetics and forces involved in the Spike ACE2 interactions with currently circulating variants remain unexplored.

The presented studies here are intended to decipher the kinetic and equilibrium binding mechanism of Spike variants on living cells for a better understanding of the viral infection process. We combine high-speed atomic force microscopy with single-molecule recognition force spectroscopy and molecular dynamics simulations to investigate, at single-molecule resolution, the interaction dynamics of trimeric Spike with its essential entry receptor ACE2 and the dynamic and nano-mechanical molecular properties of Spike. Using high-speed AFM we film conformational dynamics of isolated Spike trimers and complexes with ACE2. With single molecule force spectroscopy, we deduce the interaction strength and kinetics during the multi-bond formation of Spike protein binding and dissociation from ACE2. Finally, we model force-dependent lifetimes and avidities of Spike variant binding.

## Results

### Highly dynamic arc-like movement of Spike RBDs in real-time atomic force microscopy

**Fig. 1:**
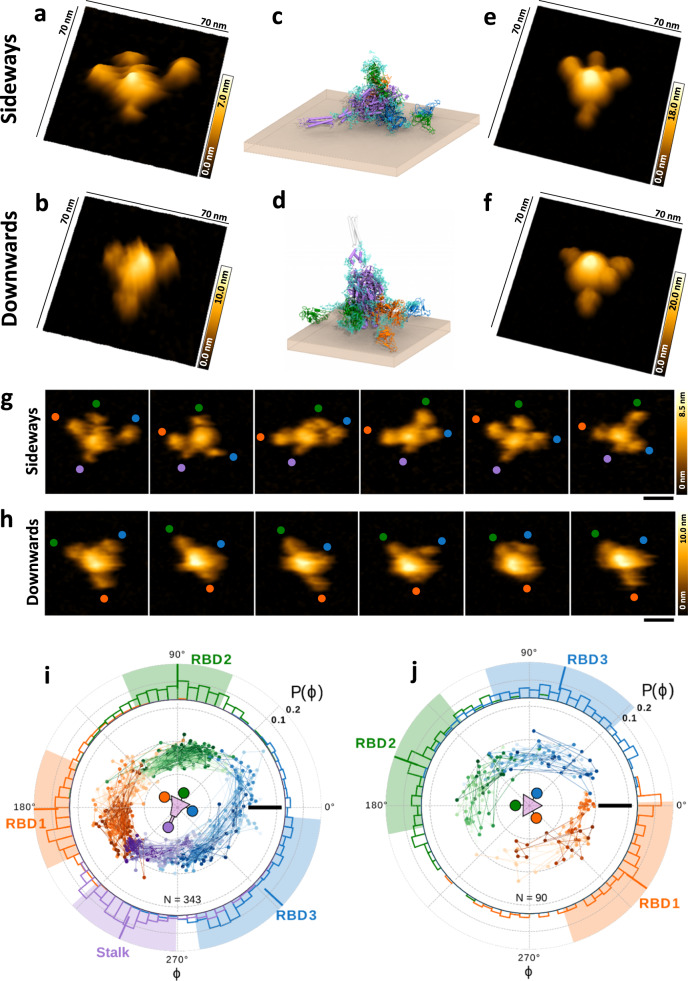
High-speed AFM analyses demonstrating the high structural flexibility of the SARS-CoV-2 Spike trimer. **a**, **b** 3D AFM image of the Spike trimer laying on mica sideways (**a**) or downwards (**b**). **c**, **d** Spike trimer model, corresponding to the conformation experimentally observed in (**a**) and (**b**), respectively. S1 domains with three RBDs are shown in orange, green, and blue. The S2 domain including the stalk is colored in purple. Glycans are shown as cyan sticks and the brown sheet represents the mica surface. The stalk is invisible in the downward configuration (**b**) and colored in faded gray (**d**). **e**, **f** 3D simulated AFM images based on the MDS models from (**c**) and (**d**). Snapshots from a movie of a single Spike trimer laying sideways (**g**) or downwards (**h**) on mica. The colored circles follow the position of the stalk (purple), and each of the RBDs (orange, green, and blue) over time. **i**, **j** Trajectories of stalk and RBDs in polar representation captured in movies (Supplementary Movies 1 and 2), corresponding to the sequences shown in (**g**) and (**h**). Radial coordinates denote the distance from the Spike center, *ɸ* is the rotational angle. Lines with increasing color intensity follow the trajectories of structures over time. The stalk is shown in purple, RBD1 in orange, RBD2 in green, and RBD3 in blue. Outer ring: The distribution of *ɸ* is shown in the colored histograms. Average values of *ɸ* are marked by a bar and the respective standard deviations by a background field in the same color. Spike orientation is schematically shown in the center. Images in **a**, **b**, **g**, and **h** were captured at a scanning speed of 154 ms/frame. Scale bar in **g** and **h**: 20 nm. Scale bar in **i** and **j**: 10 nm. Source data are provided as a Source Data file.

To elucidate the conformational flexibility of the soluble SARS-CoV-2 Spike trimer in an aqueous environment, we captured the protein assembly dynamics on mica surfaces using high-speed atomic force microscopy (AFM)^5,16–20^. We expressed and purified the soluble trimeric ectodomain of the prefusion-stabilized SARS-CoV-2 Spike protein of the Wuhan-Hu-1 reference strain, which also contained a mutated polybasic cleavage site^21^. From a series of >100 Spike trimers imaged in areas of ~100 × 100 nm^2^ and a speed of 6.5 frames per second (fps), we identified two prototypical configurations: a sideways position, with the stalk domain, tilted sideways and contacting the mica surface together with all three RBDs (Fig. 1a), and a downwards position where the Spike stalk was perpendicular to the surface and only the three RBDs were in contact with the surface (Fig. 1b). Both configurations were reproduced using molecular models based on MDS snapshots (Fig. 1c and d; see also Supplementary Fig. 1a and b for comparison of the closed and open form) and simulated AFM images derived from it (Fig. 1e and f), corresponding with published in situ cryo-ET images^10^.

The high-speed AFM imaging conditions used here allowed for the determination of lateral and rotational displacement speeds of the RBDs and did not result in any noticeable sample dragging of the scanning AFM tip. Dynamic conformational changes of the Spike trimer structures were filmed and quantified with 154 ms time resolution (Fig. 1g–j). The highly dynamic movement and flexibility of the Spike trimer captured in our high-speed AFM movies (Supplementary Movies 1–5) revealed a fast arc-like displacement of the three fully opened RBDs. The RBDs extended from the central axis of the Spike by 10–20 nm (Supplementary Fig. 2a) and exhibited an average lateral surface displacement velocity of 40–45 nm/s (Supplementary Fig. 2b). Orientation analysis showed that the RBDs rotate dynamically (Fig. 1i and j) with rotational displacement speeds of 19°–24° per 154 ms (Supplementary Fig. 2c). Depending on the sideways or downwards orientation of the Spike trimer on mica, the total range movement of all three RBD almost covered the full circular range of 360°, roughly divided into three 120° shares and with occasional small overlaps in the positions adopted by neighboring RBDs (Fig. 1i and j). The observed deviations from a perfect tripartite symmetry depend on the exact configuration of the Spike on the mica surface, possible rotations of the Spike core, and stochastic interactions of the RBDs with mica. The total displacement angles of individual RBDs were calculated as 160 ± 18° (*n* = 9) for the downwards (Fig. 1j, Supplementary Movies 3–5) and as 142 ± 18° (*n* = 6) for the sideways (Fig. 1i, Supplementary Movies 1, 2) orientation. Our results demonstrate a significantly broader opening of the RBDs (distance from RBD center of mass; residues 335–466 and 491–526; to the center of mass of the top of the central helices; residues 977–992: 102 ± 7 Å (*n* = 3) for the downwards and 95 ± 12 Å (*n* = 3) for the sideways orientation, cf. Fig. 1c, d) when compared with cryo-EM studies^1,6,8,9,22,23^ (distance from RBD center of mass to the center of mass of the top of the central helices: 48 ± 2 Å for the open (*n* = 23) and 28 ± 1 Å for the closed conformation (*n* = 34); values evaluated based on PDBids: 6vsb, 6x2b, 6xm0, 6xm3, 6xm4, 6zgg, 6zp7, 7a93, 7bnn, 7ddn, 7dk3, 7eaz, 7eb0, 7eb5, 7kdl, 7kec, 7kj5, 7lwp, 7t76, 7tgx) and all-atom molecular dynamics studies^24^. Overall, the high-speed AFM data identify the Spike protein, as well as its three RBDs, as a highly flexible and dynamic structure, permitting it to efficiently move on surfaces.

### Spike rapidly establishes up to three dynamic bonds with ACE2 in a time-dependent manner

**Fig. 2:**
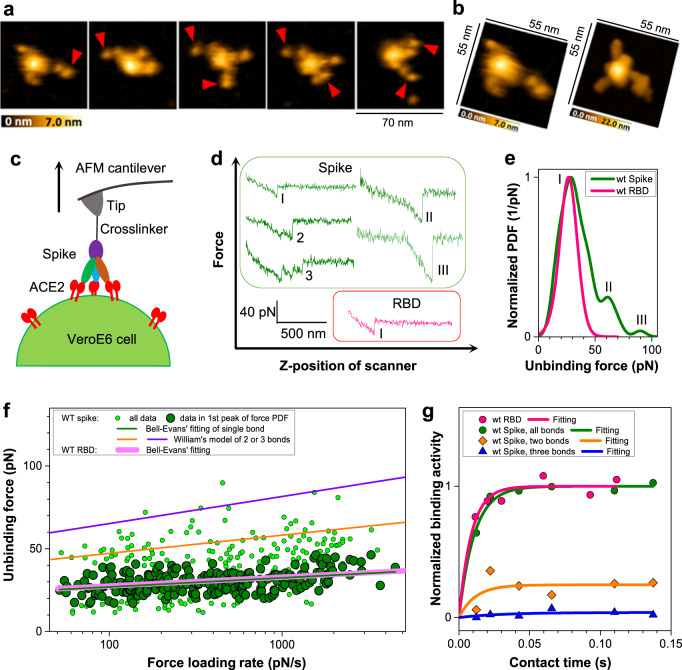
Binding of the Wuhan-1 reference strain Spike trimer and monomeric RBD to ACE2. **a** Spike trimer in complex with one or two ACE2 dimers (indicated by red arrows). **b** AFM image (left) and simulation using MDS modeling (right) of Spike trimer bound to one ACE2. **c** Schematic illustration of single-molecule force spectroscopy experiments, in which dissociation forces between the Spike trimer (or RBD) on AFM tips and membrane-anchored ACE2 expressed on VeroE6 cells were measured. **d** Exemplary force–distance curves from the experiments. The Spike trimer (green lines) exhibits up to three interactions per force curve. Multiple bonds either break sequentially ((2) and (3)) or simultaneously ((II) and (III)). Monomeric RBD shows only one interaction per force curve (red line). **e** Experimental probability density functions (PDF) at a fixed pulling velocity. Force maxima for the Spike trimer (green curve) correspond to the simultaneous dissociation of one (I) two (II) or three (III) bonds. Monomeric RBD (red curve) shows only one force maximum, reflecting the dissociation of a single bond. **f** Dynamic force spectroscopy from experiments at different pulling speeds. Dependence of dissociation forces on force loading rate. Data of the first peaks were fitted with the Bell–Evans model (thin green line), which assumes that a sharp single energy barrier is crossed for dissociation and yields the kinetic off rate, *k*_off_, and the dissociation path length, *x*_B_ (Table 1). Using the fitting parameter of the Bell–Evans theory, the orange line and blue line were calculated using the Markov binding model for uncorrelated failure of two and three bonds, respectively. The thick magenta line is the Bell–Evans fit of raw force data for RBD. **g** Dependence of binding activity on preset contact (interaction) time between the AFM tip and cellular surfaces for a Spike trimer (green circles) and for a single RBD (pink circles). Binding activity ratios for two (orange diamonds) and three (blue triangles) bonds of Spike trimer are also shown. Kinetic on-rates are retrieved from exponential fittings using pseudo-first-order kinetics of a bimolecular reaction (solid lines). Source data are provided as a Source Data file.

**Fig. 3:**
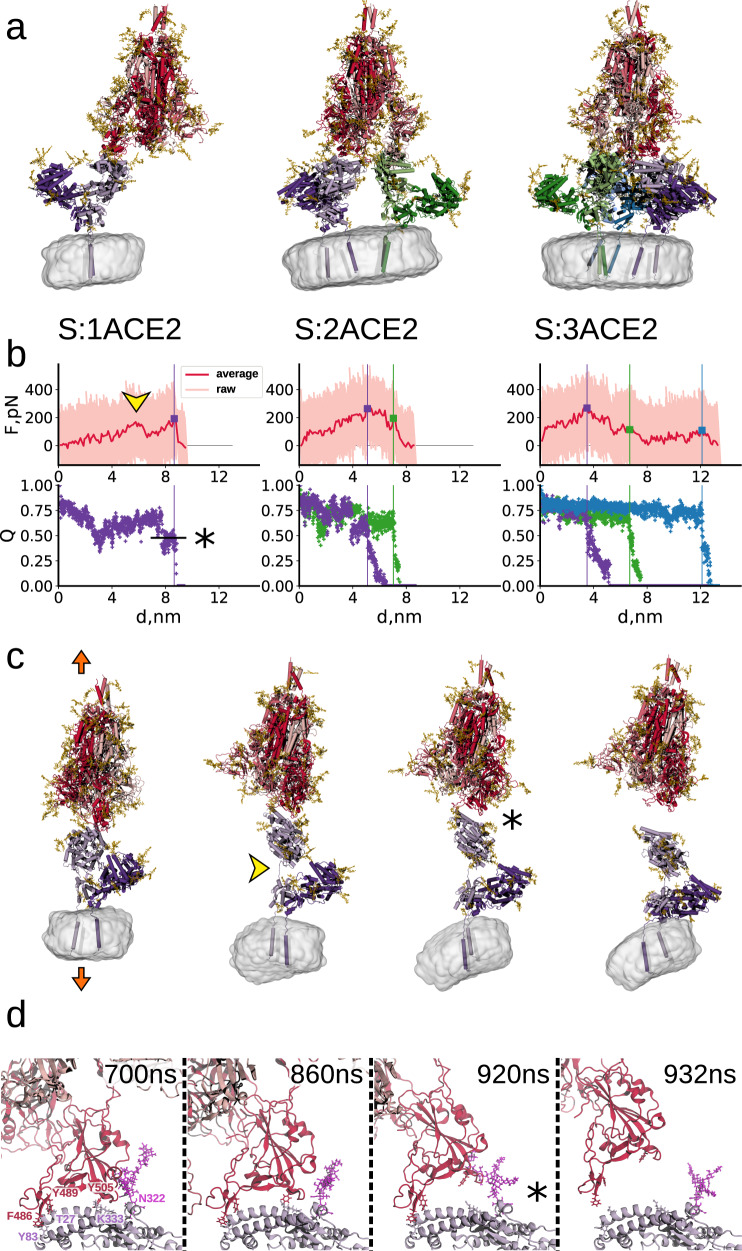
Constant velocity pulling simulations of Spike:ACE2 complexes. **a** Simulations of one, two, and three dimeric ACE2 receptors (left to right; shades of violet, green, blue; intracellular domain not shown) bound to a single Spike trimer (shades of red). The nanodisc membrane is shown in gray. Glycans on Spike and ACE2 are shown as gold sticks. **b** Force-extension (*d*) curves (top; 10-ns window average in solid red, raw data in pink) and fraction Q of Spike:ACE2 contacts (bottom). Vertical lines, yellow arrow, and star indicate dissociation events, the partial unfolding of the ACE2 linker domain, and the intermediate state with ~50% of the contacts, respectively. **c** Snapshots from the Spike:ACE2 simulation at a force loading rate of 0.0166 N/s (orange arrows, other symbols as in **b** left) until detachment (right). **d** Zoom-in on the Spike:ACE2 interface of (**c**). Key residues are indicated in red (RBD) and violet (ACE2, including its N322 glycan involved in RBD binding). The asterisk indicates the intermediate state of (**b**) and (**c**). Source data are provided as a Source Data file.

We then filmed Spike:ACE2 complexes with the high-speed AFM and visualized dynamic features of the Spike interaction with ACE2. The Spike trimer dynamics imaged in the presence of soluble, dimeric ACE2 (human ACE2 receptor ectodomain that dimerizes via its collectrin domain (amino acid 18-740)) showed that ACE2 associates with and dissociates from open RBDs in a highly dynamic fashion (Fig. 2a, b; Supplementary Movie 6;). To elucidate the strength of molecular binding between trimeric Spike and dimeric ACE2 on the single-molecule level, we performed single-molecule force spectroscopy (SMFS) experiments^25–27^ and measured dissociation forces between Spike and ACE2. Wild-type VeroE6 cells endogenously expressing the ACE2 receptor at high level^28,29^ were probed in a physiological setting with the Spike trimer or monomeric RBD coupled to AFM cantilever tips (Fig. 2c). There, the Spike trimer or monomeric RBD was conjugated to an AFM tip using a polyhistidine-tag, located opposite from the ACE2 binding site, via a 6 nm-long flexible polyethylene glycol (PEG) linker. This linkage enabled full functionality and molecular flexibility for unconstrained binding to membrane-anchored ACE2 on VeroE6 cells. Consecutive force–distance cycle measurements were performed, in which Spike:ACE2 bond formation was allowed upon approaching the Spike-loaded cantilever towards the cells. By subsequently moving the AFM cantilever away from the cellular surface with a defined speed, the formed Spike:ACE2 bonds were mechanically pulled with an external force that increased over time until they were broken. At rupture, the dissociation forces were recorded (Fig. 2d) and used to construct the experimental probability density functions of forces (Fig. 2e). The Spike trimer acted as a molecular caliper and formed up to three individual bonds (Fig. 2e) with three different ACE2 receptors. Upon pulling, they dissociated either sequentially (Figs. 2d; 2, 3) or simultaneously (Fig. 2d; II, III)^26^. In contrast, AFM tips functionalized with monomeric RBD showed only one bond breakage (Fig. 2d, e). The dissociation forces of single Spike:ACE2 bonds (~25 pN) matched those of monomeric RBD (Fig. 2d, e).

To decipher the dynamic features of the Spike:ACE2 bonds, we varied the pulling speed in our SMFS experiments. Individual dissociation forces were plotted versus their loading rate (pulling speed times effective spring constant of molecules and AFM cantilever). SMFS data of RBD and the single forces of the Spike protein overlapped and showed similar fitting results using the Bell–Evans model^30^ (Fig. 2f) for the kinetic off-rate, *k*_off_, i.e *k*_off_ = 3.2 × 10^−4±0.5^ s^−1^ for RBD and *k*_off_ = 5.2 × 10^−4±0.4^ s^−1^ for the Spike protein. Equally similar were kinetic on-rates, *k*_on_, i.e. *k*_on_ = 1.0 × 10^5±0.08^ M^−1^ s^−1^ for RBD and *k*_on_ = 0.83 × 10^5±0.04^ M^−1^ s^−1^ for the Spike trimer. The kinetic on-rates, *k*_on_, were computed from the increase of the binding activity *P*(*t*) with the tip-cell surface contact time *t*_c_ (Fig. 2g), by approximating with pseudo-first-order kinetics according to *P* = *A*(1−exp(−(*t*_c_−*t*_0_)/*τ*)), and calculated using *k*_on_ = 1/*τC*_eff_(*C*_eff_, effective concentration of molecules coupled to the AFM tip)^26^. The kinetic binding data were in line with SPR experiments, in which binding of ACE2 to surface-coupled Spike trimer was followed over time (Supplementary Fig. 3). Affinities computed from the kinetic rates resulted in a *K*_D_ = 3.2 × 10^−9±0.5^ M for RBD and K_D_ = 6.2 × 10^−9±0.5^ M for a single RBD of the Spike trimer (*p* = 0.56). As a control, soluble ACE2 blocked Spike binding to host cell membranes as bi-valent inhibitor (Supplementary Fig. 4a, b); the IC_50_ value (0.38 nM) of inhibition by soluble ACE2 was markedly superior to the affinity constant derived for single RBD:ACE2 bonds (6.2 nM). Of note, soluble ACE2 can effectively neutralize SARS-CoV-2 binding to host cells^14,15,31^ and is currently undergoing clinical tests for the treatment of Covid-19.

We next analyzed the behavior of multiple bonds formed between the Spike trimer and ACE2. Binding of a single Spike to two or three ACE2 molecules via the second and third RBD exhibited a ~2- and 3-fold higher resistance to dissociation, respectively, when compared to a single bond (Fig. 2d, e). In comparison with the binding of the first RBD to ACE2, the probability for binding of the second and third RBD consecutively decreased (Fig. 2e, g, Table 2), indicating that individual RBD entities bind to ACE2 sequentially. The time course for the formation of the second and third bond (Fig. 2g) resulted in on-rates of 8.7 × 10^1±0.3^ and 3.2 × 10^1±0.4^ s^−1^ (Table 2), respectively, revealing that the association kinetics for the third RBD to ACE2 is more than two times slower than for the second RBD, most likely, because of steric restrictions. Successive bond formation occurred within about 100 ms. The kinetic off-rate data of single Spike:ACE2 bonds were fitted with the Bell–Evans theory (Fig. 2f). Based on these fitting parameters, a Markov binding model computed the theoretical forces for the dissociation of two and three bonds. The uncorrelated dissociation mode underlying this model implies that there is no particular mechanical coupling between the bonds. A good match of this model with the off-rate data for double- and triple-bond rupture of Spike from ACE2 (Fig. 2f) revealed independent dissociation of the three RBDs. Both association and dissociation modes of the Spike RBDs to ACE2 on cellular surfaces, here explored at the single-molecule level, must arise from the high flexibility of the linker domains connecting the RBDs to the core helices of the Spike trimer^32^. This assumption is consistent with the dynamics we observed in the high-speed AFM videos (Supplementary Movies 1–5). Collectively, our single-molecule force spectroscopy data show that the SARS-CoV-2 Spike from the Wuhan-1 reference strain displays a sequential binding mode via its RBDs to three ACE2 molecules, thereby establishing enhanced binding avidity.

### Amino acid contacts assisted by transient ACE2 glycan binding establish the Spike:ACE2 bond strength

To identify dominant binding interfaces in the molecular mechanism of the Spike:ACE2 dissociation process, we performed constant velocity pulling MDS with soluble trimeric Spike and ACE2 embedded in nanodisks (Fig. 3a, Supplementary Movies 7–9). Consistent with the AFM results using ACE2-expressing VeroE6 cells (Fig. 2d), we observed both sequential and simultaneous dissociation of the RBDs in a Spike trimer from one, two, or three dimeric ACE2 receptors embedded in nanodisks. Sequential dissociation was more frequently observed at this nanosecond time resolution (Fig. 3b, top panels). A drop in force was associated with the loss of native contacts (Q, see Online Methods) at the different Spike:ACE2 interfaces (Fig. 3b, bottom panels). This allowed us to assign the dissociation force to distinct interfaces, with a marked increase of the forces at higher loading rates and no correlation between the order of Spike:ACE2 dissociations and the force (Supplementary Fig. 5, 6, and 7). Interestingly, the linker domain between the dimerization and peptidase domains of ACE2 (residues 565–590) partially unfolded (interdomain distance > 0.55 nm, see Supplementary Fig. 8) and quickly refolded following the Spike:ACE2 bond rupture. A transient increase of the force was associated with the unfolding (Fig. 3b, c), which was however not captured by the fraction of Spike:ACE2 contacts (Fig. 3b), as the unfolding occurs at a relatively large distance from the Spike:ACE2 interface. In the MD simulations, the drop in force after a bond-rupture event is smaller than in the experiments. This is a result of the faster-pulling speed, which leaves the system less time to relax, and because of the absence of the tether in the experiments, which softens the force response.

Both the ACE2 peptidase domain and the RBD, however, remained stably folded during pulling (Fig. 3c, d; Supplementary Fig. 8). The two-step dissociation process of the Spike:ACE2 interface started with a detachment of the Y505^S^:K353^ACE2^ (upper index denotes the host protein for given amino acids) flank (Supplementary Figs. 9 and 10). The distance between these two residues increased to ~1 nm until the other flank (F486^S^,Y489^S^:T27^ACE2^,Y83^ACE2^) was compromised, which caused the whole Spike:ACE2 interface to fully dissociate (Fig. 3d and Supplementary Figs. 11 and 12). The opening of this interface together with the elongation of the ACE2 linker domain could explain the particularly long dissociation path determined from the pulling experiments (*x*_B_ = 1.7 nm; Table 1). As soon as the Y505^S^:K353^ACE2^ flank detached, the dissociation process quickly proceeded with a lag time in the range of 0–200 ns (Supplementary Fig. 12).

**Table 1:** Summary of kinetic rate constants (*k*_on_, *k*_off_), width of energy barriers (*x*_B_), and affinities (*K*_D_) of SARS-CoV-2 Spike or RBD of Wuhan reference strain, defined RBD mutants, and variants of concern determined using single molecule force spectroscopy

	Wuhan reference strain spike	Spike mutant N234Q	Delta variant spike	Omicron variant spike	Wuhan reference strain RBD	RBD mutant N501Y	RBD mutant E484K	Delta variant RBD	Omicron variant RBD
*k*_on_ (M^−1^ s^−1^)	8.3 × 10^4±0.1^	1.1 × 10^4±0.2^	8.9 × 10^4±0.1^	7.4 × 10^4±0.1^	1.0 × 10^5±0.1^	7.6 × 10^4±0.1^	4.7 × 10^4±0.1^	6.6 × 10^4±0.1^	7.1 × 10^4±0.2^
*x*_B_ (nm)	1.7 ± 0.25	1.6 ± 0.43	1.9 ± 0.13	2.0 ± 0.21	1.7 ± 0.25	1.9 ± 0.26	2.0 ± 0.24	1.9 ± 0.26	2.0 ± 0.10
*k*_off_ (s^−1^)	5.2 × 10^−4±0.4^	4.0 × 10^−4±0.6^	4.4 × 10^−5±0.3^	4.1 × 10^−6±0.5^	3.2 × 10^−4±0.5^	4.8 × 10^−5±0.5^	5.9 × 10^−5±0.4^	6.4 × 10^−5±0.5^	4.2 × 10^−6±0.2^
*K*_D_ (M)	6.2 × 10^−9±0.5^	3.6 × 10^−8±0.7^	5.0 × 10^−10±0.3^	5.5 × 10^−11±0.2^	3.2 × 10^−9±0.5^	6.3 × 10^−10±0.5^	1.3 × 10^−9±0.5^	9.7 × 10^−10±0.5^	6.8 × 10^−11±0.2^

Amino-acid contacts (~25 between one Spike trimer and one ACE2 protein) accounted for the majority of interactions within the binding interface (Supplementary Fig. 13). The second largest contribution came from the interaction of ACE2 glycans with Spike, among which N53, N90, and N322 have been described to contribute to the Spike:ACE2 interaction^12,14,32,33^. The ACE2 N322 glycan had the highest number of contacts with the Spike protein until the very end of the unbinding process in most of our MDS setups. Interestingly, in several cases this glycan transiently re-bound to Spike and thus restored the interface after the protein–protein contacts were almost fully severed (Supplementary Fig. 13). By contrast, Spike glycans remained dynamic, mainly detached from ACE2, and, consistent with the previous work^32^, formed only very transient contacts with ACE2 glycans and none with the ACE2 protein backbone.

### The key role of the N234Q glycosite on Spike dynamics and bond formation with ACE2

**Fig. 4:**
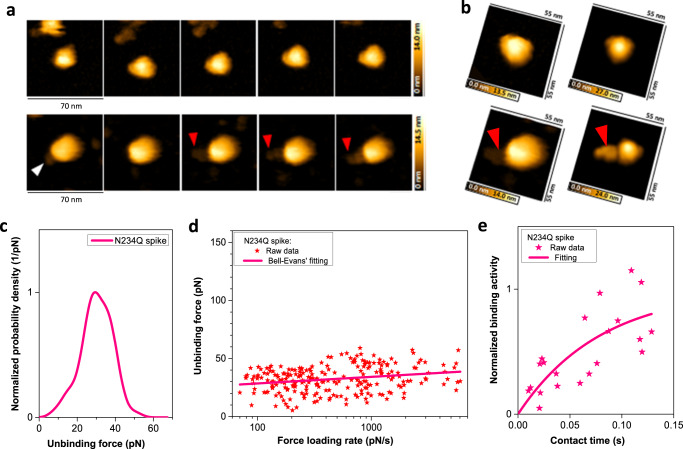
Binding of Spike N234Q glycomutant to ACE2. **a** First row: Single Spike trimer N234Q mutant imaged using high-speed AFM. No details of RBDs or stalk are visible and only small conformational changes were observed. Second row: Single Spike trimer N234Q imaged in buffer solution containing free ACE2. One RBD in the open conformation (indicated by a white arrowhead) is bound to ACE2 (red arrowhead). **b** Left panels: AFM images of a single Spike mutant N234Q without ACE2 (upper image) and complexed with ACE2 (lower image). Right panels: simulations of a closed Spike trimer without ACE2 (upper image) and complexed with ACE2 (lower image). **c** Spike trimer N234Q shows only one force maximum, reflecting the dissociation of a single bond. **d** Dependence of dissociation forces on the force loading rate at different pulling speeds. Solid line is the Bell–Evans fit of dissociation force data (red stars) for Spike trimer N234Q. **e** Dependence of binding activity on contact time between AFM tip and cellular surfaces for the Spike trimer N234Q (red stars). Solid line represents fittings using pseudo-first-order kinetics of a bimolecular reaction. Source data are provided as a Source Data file.

Given the importance of the Spike glycan shield in host immune defense^1,34–36^ and structural stabilization^37,38^, we set out to assess the binding dynamics of a Spike trimer carrying a mutant N234—glycan site. Previous MDS reported this glycan to be important for maintaining the RBDs in the open state^24,36,37^ and bilayer interferometry (BLI) experiments^37^ revealed that ablation of the N234 glycan significantly reduced binding of the Spike protein to ACE2. Importantly, our high-speed AFM recordings of the Spike N234Q glycomutant trimer showed that, in striking contrast to the Wuhan-Hu-1 reference strain, the RBDs of the Spike N234Q was much less dynamic and were predominantly in a closed downwards configuration (Fig. 4a, b, first rows, Supplementary Movie 10), with only a transient slight opening of a single RBD (Fig. 4a, b second rows, Supplementary Movie 11). Spike N234Q formed only single bonds with ACE2 (Fig. 4c, Table 2) expressed on the surface of VeroE6 cells. Interestingly, the binding strength and kinetic off-rate, *k*_off_ = 4.0 × 10^−4±0.6^ s^−1^ (Fig. 4d), were comparable between the reference Spike protein and the Spike glycomutant N234Q. Yet, the kinetic on-rate of Spike N234Q (Fig. 4e; *k*_on_ = 1.1 × 10^4±0.2^ M^−1^ s^−1^) was significantly lower than what we observed for the reference strain Spike trimer (8.3 × 10^4±0.04^ M^−1^ s^−1^; *p* < 0.05; Fig. 2g), resulting in a markedly decreased *K*_D_ of 3.6 × 10^−8±0.7^ M (Table 1). Collectively, these results reveal that the Spike conformational freedom and dynamics are key in maintaining the kinetics of bond formation and the capability for establishing multiple bonds with the ACE2 receptor.

**Table 2:** Percentage and on-rate of two or three bonds from Spikes of Wuhan reference, N234Q, Delta, and Omicron (B.1.1.529) variant

	Wuhan reference	N234Q mutant	Delta variant	Omicron variant
Percentage of one bond	70.3	100	68.7	85.4
Percentage of two bonds	27.3	0	26.0	13.6
Percentage of three bonds	2.4	0	5.3	1.0
On-rate of two bonds (s^−1^)	8.7 × 10^1±0.3^	–	8.0 × 10^1±0.2^	4.9 × 10^1±0.2^
On-rate of three bonds (s^−1^)	3.2 × 10^1±0.4^	–	2.3 × 10^1±0.3^	1.1 × 10^1±0.4^

### Delta and Omicron (B.1.1.529) Spike form bonds of markedly increased strength with ACE2

**Fig. 5:**
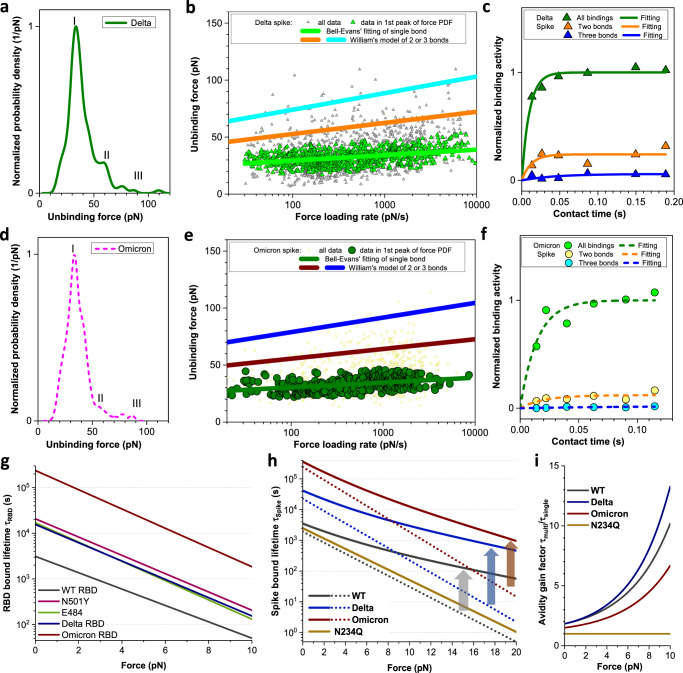
Mutations and avidity amplification lead to the prolongation of Spike binding to ACE2. **a**, **d** Experimental PDFs from measured dissociation forces between Spike and ACE2. Force maxima for the Spike trimer Delta (**a**) and Omicron (B.1.1.529) (**d**) variants correspond to the simultaneous dissociation of one (I), two (II), or three (III) bonds. **b**, **e** Dependence of dissociation forces on the force loading rate. Data of the first peak (**b**, green triangles for Delta variant and **e** olive circles for Omicron (B.1.1.529)) were fitted with the Bell–Evans theory (**b**, green line for Delta variant and **e**, olive line for Omicron (B.1.1.529)). The Markov binding model for two and three bonds are computed and shown as orange and cyan lines for the Delta Spike (**b**) and wine and blue lines for Omicron (B.1.1.529) Spike (**e**), respectively. **c**, **f** Dependence of binding activity on contact time between AFM tip and cellular surfaces for the Delta Spike (**c** olive triangle) and the Omicron (B.1.1.529) Spike (**f** green circles). Ratio of force curves containing two (**c**, orange triangles for Delta and **f**, yellow circles for Omicron (B.1.1.529) Spikes) and three (**c** blue triangles for Delta and **f** cyan circles for Omicron (B.1.1.529) Spikes) bond breakages are shown and fitted with pseudo-first-order kinetics (**c** for Delta and **f** for Omicron (B.1.1.529) Spike; orange lines for two bonds and blue lines for three bonds). **g** Lifetime of the RBD:ACE2 complex under force for the Wuhan reference strain (WT), the indicated mutants, and variants. **h** Lifetime of the Spike:ACE2 complex at increasing external force for WT and the indicated variants. Dotted or solid lines represent one RBD or full trimeric Spike. Arrows (gray for WT, blue for Delta, brown for Omicron) indicate lifetime increase arising from multivalent Spike binding. **i** Ratio of bound lifetimes for RBD triple bonds compared to individual RBD bonds, termed as avidity gain factor *τ*_multi_/*τ*_1Spike_. The lifetimes of WT and the indicated variant Spikes were calculated according to the kinetic model of Williams except for N234Q, where only a single RBD binds. Source data are provided as a Source Data file.

Next, we aimed to assess the impact of defined receptor binding domain mutations shared among the Spike proteins of several SARS-CoV-2 variants of concern (VOC). In this respect, we focused on N501Y, which is a common RBD mutation present in the Alpha, Beta, and Gamma variants, as well as E484K, which is carried by the vast majority of Beta and Gamma variants. Further, we wanted to elucidate differences in the ACE2 binding kinetics between the recent Delta (mutations L452R, T478K, P681R) and the Omicron (B.1.1.529 variant; mutations A67V, HV69-70del, T95I, G142D, 306 VYY143-145del, N211del, L212I, ins214EPE, G339D, S371L, S373P, S375F, K417N, N440K, 307 G446S, S477N, T478K, E484A, Q493R, G496S, Q498R, N501Y, Y505H, T547K, D614G, 308 H655Y, N679K, P681H, N764K, D796Y, N856K, Q954H, N969K, L981F) VOC Spike proteins as compared to the Wuhan isolate^39–42^. The binding strengths of the N501Y and E484K RBD mutants and the Delta Spike trimer were significantly higher and displayed almost 10-fold lower kinetic off-rates (for Delta Spike *k*_off_ = 4.4 × 10^−5±0.3^ s^−1^, Fig. 5b, Supplementary Fig. 14a, c, e) as compared to that obtained with the reference strain (*k*_off_ = 5.2 × 10^−4±0.4^ s^−1^, *p* < 0.001). Thus, the markedly increased binding of the Delta variant might contribute to its increased infectivity^43^. Amazingly, the kinetic off-rate of the Omicron (B.1.1.529) Spike trimer was 100-fold lower (*k*_off_ = 4.2 × 10^−6±0.1^ s^−1^, *p* < 0.05, Fig. 5e) compared to the reference strain. In contrast, with both tested Spike variants we did not observe differences in the kinetic on-rates (for Delta Spike 0.89 × 10^5±0.1^ M^−1^ s^−1^, for Omicron (B.1.1.529) Spike 0.74 × 10^5±0.1^ M^−1^ s^−1^, Fig. 5c, f; Supplementary Fig. 14b, d, f). The stable ACE2 attachment to the Delta and Omicron (B.1.1.529) Spike trimer was confirmed in high-speed AFM images (Supplementary Fig. 15a, b, Supplementary Movies 12, 13) and may ultimately result in an increased probability for viral entry.

Consistent with the single-molecule force spectroscopy experiments results obtained with the Spike protein of the reference strain (Fig. 2), we also observed double and triple bond formation with the Spike trimer of the Delta variant on VeroE6 cells expressing ACE2 (Fig. 5a, b, Table 2) with high specificity (Supplementary Fig. 16a). Binding kinetics of the second (8.0 × 10^1±0.2^ s^−1^ vs. 8.7 × 10^1±0.3^ s^−1^) and third (2.3 × 10^1±0.4^ vs. 3.2 × 10^1±0.6^ s^−1^) RBD of the Delta Spike (Fig. 5c) were comparable to that of the reference strain. In striking contrast, the Omicron (B.1.1.529) Spike formed fewer double and hardly any triple bonds with ACE2 (Fig. 5d–f, Table 2), and the binding rates for the second and third RBD were slow (4.9 × 10^1±0.1^ and 1.1 × 10^1±0.4^ s^−1^, respectively, Fig. 5f). This finding was unexpected, considering its high specificity (Supplementary Fig. 17) and 100-fold increased affinity (Table 1) for single bond formation with ACE2 when compared with the reference strain. These findings correlate with high-speed AFM images of the Delta and Omicron (B.1.1.529) Spike Trimer (Supplementary Movies 14–19) and the RBD displacement analysis derived thereof (Supplementary Fig. 2). While the behavior of the Delta Spike was similar to that of the Wuhan reference strain, the Omicron (B.1.1.529) variant showed less pronounced RBD opening, accompanied by reduced angular and lateral displacements.

### Mutations and avidity amplification lead to prolongation of Spike binding to ACE2

To quantify the effect of the multiple-bond strength of the Spike:ACE2 interactions on avidity, we applied a model based on the theories of Bell^44^, Evans^30^, and Williams^45^, in agreement with our data for single and multiple bond ruptures (Figs. 2f, 4d, and 5b,e). With this model, we calculated force-dependent lifetimes for monovalent binding of the RBDs (*τ*_RBD_, Fig. 5g) and for multivalent binding of the trimeric Spike protein (*τ*_Spike_, Fig. 5h). Accordingly, a trivalent binding of the Wuhan reference strain and the Delta Spike variant, a bivalent binding of the Omicron (B.1.1.529) Spike variant, and a monovalent binding of the N234Q Spike mutant was assumed (Table 2). We then expressed the effect of multivalent binding as avidity gain factor *τ*_multi_/*τ*_1Spike_ by comparing multiple vs. one Spike-RBD:ACE2 interactions (Fig. 5i). Both RBD mutations N501Y and E484K and mutations carried by the Delta and Omicron (B.1.1.529) variants of concern (Fig. 5g), as well as the increase in avidity arising from the multiple-bond formation (Fig. 5i) were associated with a significant increase in overall interaction lifetime (Fig. 5h). The combined effects of avidity and mutations within the binding interface thereby determine the dependence of the lifetime on the force exerted to the Spike:ACE2 bonds (Fig. 5h). At zero force, the bond lifetime of the bound Spike Omicron (B.1.1.529) variant is almost 10-fold and more than 100-fold higher than that of the Delta variant and the Wuhan reference strain, respectively (3.7 × 10^5^ vs. 4.2 × 10^4^ vs. 3.5 × 10^3^ s). Yet, the bond lifetimes of the reference strain and Delta Spike display a less steep decrease with increasing external forces due to the force-induced avidity gain by triple-bond formation with ACE2 (Fig. 5i). In contrast, the Spike Omicron (B.1.1.529) variant forms only double bonds with ACE2 and its bond lifetime decreases more drastically with applied external force. The force exerted onto a virus particle of ~80 nm diameter^10^ by the cilia-driven mucus flow on lung epithelial cells is in the range of 20 pN^46,47^. At this force, the bond lifetimes of the Spike Omicron (B.1.1.529) and Delta are similar (960 and 460 s, respectively) and exceed the bond lifetime of the Spike Wuhan reference strain by one order of magnitude (56 s) (Fig. 5h). Consequently, the prolonged binding of the Omicron (B.1.1.529) and Delta variant Spike is expected to increase the chance of successful viral cell entry and infection.

## Discussion

Our data show that the Spike trimer of the Wuhan-1 reference strain and the Delta variant is tailored for three-valent attachment to three different ACE2 molecules expressed on living cells. High-speed AFM imaging revealed that both proteins are highly flexible and dynamic structures, which possess three rapidly moving open RBDs. The flexibility of the host cell receptor ACE2 and the clustering of Spike on the viral surface^10^ might additionally aid in establishing multiple bonds, which are stabilized by ACE2 glycans at the Spike:ACE2 binding interface. In line with our findings, it was recently reported that the SARS-CoV-2 Spike ectodomain of the Wuhan strain reversibly samples open-trimer conformations^48^ in solution at ambient conditions. In contrast, the Spike trimer carrying a mutant N234—glycan site appeared predominantly in a closed downwards configuration, with restricted RBD movement and only a transient slight opening of a single RBD. As such, this glycosite mutant also contributes as a negative control for our high-speed AFM imaging conditions that do not per se force the RBDs to open. In addition, the Spike N234Q glycomutant trimer is an excellent model to highlight the importance of the Spike’s conformation and dynamics on the kinetics of bond formation and the potential for three-valent attachment. Our single-molecule force spectroscopy experiments showed that Spike N234Q trimer bound only monovalently to ACE2 molecules, with an almost 10 times slower kinetic on-rate when compared to the Wuhan-1 reference strain and the Delta variant.

The Delta Spike variant shows enhanced and prolonged attachment to ACE2 when compared to the Wuhan-1 reference strain. It has been reported that enhanced electrostatic interactions at the RBD-ACE2 interface lead to an increase in affinity in BLI experiments^49^. Our single molecule force spectroscopy studies on living cells reveal off rates reduced by one order of magnitude, which implies markedly enhanced resistance against detachment by external forces typically exerted on viral particles. It has been found that Delta Spike can fuse membranes more efficiently at low levels of cell surface ACE2 expression and Delta Spike-pseudotyped viruses infect target cells substantially faster than other SARS-CoV-2 variants^50^.

The Omicron (B.1.1.529) variant Spike displays a strikingly different nanomechanical binding mode than the Wuhan-1 reference strain and the Delta variant. On one hand, the Omicron (B.1.1.529) RBD has a mutational profile that allows for improved binding to ACE2^51–54^ as compared to the Wuhan-1 or Delta Spike. This is confirmed by our data (Table 1) and is consistent with BLI^52,54^ and SPR experiments^52^ performed by others. We found that Omicron (B.1.1.529) RBD binding strength and affinity for ACE2 were even more enhanced when compared to that of the Delta variant (factor of 10), providing a potential explanation for the higher infectivity of the Omicron (B.1.1.529) variant^51^. On the other hand, the ability of the Omicron (B.1.1.529) Spike to form multiple bonds with ACE2 was markedly decreased, leading to a loss in avidity amplification. This correlates with our high-speed AFM observations, showing less pronounced RBD opening and reduced angular and lateral RBD displacements, and is also consistent with recent EM studies reporting steric restrictions arising from a binding interface between two adjacent RBDs^51,52,55,56^ that may lead to tight packing of the Omicron (B.1.1.529) RBDs^57^ in the 3 RBD-down states and a slower transition of the Omicron (B.1.1.529) Spike from one-RBD-up to two-RBD-up or three-RBD-up conformations^51^. Furthermore, our data is also in line with a previous study demonstrating that a one-up RBD conformation of the Omicron (B.1.1.529) Spike is preserved after ACE2 binding, which is in stark contrast to other VOCs, where ACE2 binding of one RBD triggers the exposure of further up-RBDs^58^.

The RBD transitions are a key trigger for S1 dissociation and S2 refolding and are required for bringing the viral and host membranes in close proximity^59^ for membrane fusion^51^. It has also been reported that suboptimal cleavage at the S1/S2 site^51^, even in cells endogenously expressing the membrane-anchored serine protease TMPRSS^60^, results in decreased fusogenicity of the Omicron (B.1.1.529) Spike^51^, compared to Delta. This consequently leads to a marked reduction in cell entry via TMPRSS2-supported plasma membrane fusion and viral replication in TMPRSS2-expressing cells^53,61^. Nonetheless, Omicron (B.1.1.529) enters cells more efficiently in a TMPRSS-independent manner^54,62^ via the endosomal route by Spike cleavage through endosomal cathepsin^62^. A possible reason might be the lack of interaction with the membrane-anchored TMPRSS^63^ due to the ACE2 binding mode of the Omicron (B.1.1.529) Spike. Hereby, Omicron (B.1.1.529) infects a greater number of cell types in the respiratory epithelium, resulting in enhanced transmissibility^61,64^.

Our data using real-time cell-based atomic force microscopy uncover the binding modes of the variant Spike molecules to ACE2 and provide a nanomechanical explanation of increased viral infection and transmissibility of recent and current variants of concern. In particular, our results provide nanoscale insights into the unique capacity of Omicron (B.1.1.529) to bind to single ACE2 molecules with exceedingly strong binding affinity, which might explain the rapid spread of this SARS-CoV-2 variant even at very low ACE2 expression.

## Methods

### High-speed (HS)-AFM sample preparation and imaging

Purified standard strain SARS-CoV-2 Spike trimer or SARS-CoV-2 Spike trimer N234Q variant, Delta or Omicron (B.1.1.529) variant stocks were diluted to 0.2 µM in (10 mM Hepes pH 7.4, 140 mM NaCl, 5 mM KCl, 1 mM CaCl_2_, 1 mM MgCl_2_, 0.1 mM NiCl_2_). Subsequently, the solution was diluted to 10 nM in imaging buffer (10 mM Hepes pH 7.4, 140 mM NaCl, 5 mM KCl, 1 mM CaCl_2_, 1 mM MgCl_2_) and 1.5 µl of the solution was applied onto freshly cleaved mica discs (1.5 mm diameter). After 3 min, the surface was rinsed with ~1 5µl imaging buffer (without drying) and the sample was mounted into the imaging chamber of the HS-AFM^16^. For observing the complexation of ACE2 and Spike, the Spike was deposited on mica as described above, and 10 µg/ml of soluble ACE2 in imaging buffer was added into the fluid chamber prior to imaging. HS-AFM movies were recorded in the solution containing free ACE2 using the software AFM Imaging, Version 2.9’. Both, standard strain Spike and the N234Q variant, were expressed in HEK cells and provided by M.K. (Institute of Molecular Biotechnology, University of Natural Resources and Life Sciences, Vienna). The Delta and Omicron (B.1.1.529) variant Spikes were also expressed in HEK cells and obtained from AcroBiosystems. For imaging, we used the ultra-short cantilevers USC-F1.2-k0.15 (NanoWorld) with a spring constant of 0.15 N/m, resonance frequency of ~500 kHz, and quality factor of ~2 in liquid. During image acquisition, the amplitude was kept constant and set to 90–85% of the free amplitude, typically ~3 nm.

### Image processing

Image processing was done using Gwyddion 2.55^65^. Horizontal scars, occurring from feedback instabilities were selected and removed by Laplacian background substitution. A height threshold mask was used for selecting the background prior to correction of scan line artifacts and polynomial background. Next, a Gaussian filter was applied to reduce the remaining noise and improve the visualization of the molecules.

### Trajectory tracking

After drift-correction of individual HS-AFM movies using the image stabilizer plugin (available for download at www.cs.cmu.edu/~kangli/code/Image_Stabilizer) of Fiji^66^, cartesian coordinates of the three RBDs, Spike stalk, and the Spike central domain were manually tracked (tracking plugin available for download at https://github.com/fiji/Manual_Tracking) and normalized to the position of the central domain. Transformation to polar coordinates was done using custom python scripts, and polar histograms as well as circular means and circular standard deviations were calculated as implemented in SciPy (www.scipy.org). Polar histograms and time traces were plotted using Matplotlib^67^.

### Simulated AFM

Molecular models were pre-oriented and shifted such that the lowest atom coincides with the *z* = 0 plane. Next, a grid was defined spanning the dimensions of the molecule and enlarged in *x*–y to form a 100 × 100 nm square, with lattice constants of 0.5 nm in *xy* and 0.7 nm in *z*. For each grid point, C_*α*_ atoms of the molecule were tested for collision with the sphere of 2 nm radius centered at a given point (AFM tip) and a cone protruding towards +*z* with a cone opening angle of 30° for downwards and sideways configurations, and 20 degrees for downwards configuration with ACE2 bound. For each *xy* grid point, the *z*-position of the highest clashing position was taken as the height reported by the AFM image. To mimic the experimental images, Gaussian noise was added in *z*, with zero mean and standard deviation of 0.03 nm. Finally, images were convolved with a Gaussian filter, with a standard deviation equivalent to one pixel.

### Simulated AFM model generation

Initial full-length models of closed Spike trimers were obtained from Sikora et al.^68^. In these models, two of the RBDs are in a closed configuration and one RBD is partially open. To generate a model with all RBDs open, the initial structure was placed in a rectangular simulation box with dimensions 28.3 × 24.5 × 47.0 nm^3^. The long axis of Spike was aligned with the *z*-axis of the box and positioned with atom *z* coordinates >10 nm. We mimicked the adherence of Spike to the mica surface as follows. First, we defined atom groups: (i) N-terminal domain (NTR, residues 1-291); (ii) RBD (residues 335–518); (iii) top helices in the core (Spike core, residues 737–758 and 963–1002). Next, we introduced a flat-bottomed potential at *z* = 10 nm, which acted repulsively on all defined groups and prevented them from crossing the modeled “mica” surface. In each simulation, we adjusted the distance between RBDs and the Spike core by applying a harmonic umbrella potential acting in the *xy* plane between each of the RBDs and the center of mass of the Spike with a force constant of 250 kJ/(mol nm^2^). We varied the target distances between the RBDs and the Spike core from 6 to 20 nm with a 1 nm increment. Finally, to mimic the adherence of Spike to the mica surface, a harmonic potential was introduced with a minimum at *z* = 10 nm and force constants of 1, 500, and 1000 kJ/mol/nm^2^ for NTR, RBD, and Spike core, respectively. Each system was simulated in a vacuum until the desired RBD protrusion was achieved (150,000 steps, step size 4 fs). Simulation snapshots best corresponding to observed HS-AFM images were then selected for visualization. The position of one of the RBDs was further manually adjusted using Pymol in the Spike oriented sideways, to best represent the arrangement seen in the HS-AFM images. In a final step, the three RBDs were replaced with Spike RBDs in a native open conformation (residues 331–531) as extracted from PDBid 7a98 and placed with a minimum root mean square distance (RMSD) superposition. The resulting models were then used to simulate HS-AFM images.

### Spike protein dynamics quantification

Using the cartesian coordinates of RBDs, stalk, and central domains of Spike proteins determined from the HS-AFM movies, we evaluated their dynamics from image frame to frame and calculated the distances from the center, the angular displacement and the displacement speed.

The distances covered per frame (*D*) of the RBDs and the stalk were calculated by applying the following equation:

1 $$D=\,\sqrt{{({x}_{n}-({x}_{n+1}-({x}_{0n+1}-{x}_{0n})))}^{2}+{({y}_{n}-({y}_{n+1}-({y}_{0n+1}-{y}_{0n})))}^{2}}$$

where *x*_*n*_ and *y*_*n*_ are the initial positions of the end of RBD or stalk, *x*_*n*+1_ and *y*_*n*+1_ are the positions of the RBD or stalk in the subsequent frame, *x*_0*n*_ and *y*_0*n*_ are the initial positions of the center, and *x*_0*n*+1_ and *y*_0*n*+1_ are the positions of the center in the subsequent frame.

The distance from the center (*R*) of end of RBDs and stalk domains in each frame was calculated applying the equation:

2 $${R}_{n}=\sqrt{{({x}_{n}-{x}_{0n})}^{2}+{({y}_{n}-{y}_{0n})}^{2}}$$

The angular displacement of RBDs and stalk domains per frame (*θ*_d_) was calculated using:

3 $${\theta_d}={ArcCos}\, \left(\frac{{{R}_{n}}^{2}+{{R}_{n+1}}^{2}-\,{D}^{2}}{2{R}_{n}{R}_{n+1}}\right)$$

where *R*_*n*_ is the initial distance between the centers of the RBD and stalk domains and *R*_*n*+1_ the corresponding distance in the subsequent frame.

The displacement speed of RBDs and stalk domains per frame (DS) was calculated using:

4 $${{{{{\rm{DS}}}}}}=\,\frac{{D}}{{{{{{\rm{Time}}}}}}\; {{{{{\rm{per}}}}}}\; {{{{{\rm{frame}}}}}}}$$

The resulting data were plotted using the OringinPro software.

### Molecular dynamics simulations

All MDS were performed with GROMACS^69^ 2019.6 engine with the CHARMM36m force-field extended to glycans^70–72^. Unless stated otherwise, we used TIP3P water model^73^ with Luo and Roux ion parameters^74^. After solvation, the systems were energy minimized with the steepest descent algorithm (3000 steps), followed by equilibration in the NVT ensemble for 250 ps (time step of 1 fs). During equilibration, the temperature was maintained using the Berendsen thermostat^75^ (coupling constant of 1 ps). The systems were further equilibrated in an NPT ensemble (Parinello–Rahman pressure coupling^76^ \l “with a time constant of 5 ps and compressibility of 4.5 ×10^−5^ bar^−1^ with isotropic pressure coupling for all truncated systems and semiisotropic coupling for the full membrane-embedded Spike) over the course of 1.625 ns. During the equilibration, position and dihedral restraints were gradually reduced, with force constants changed from 1000 to0 kJ/(mol nm^2^). Hydrogen bonds were restrained using the LINCS algorithm^77^. Following the procedure applied before to enhance the simulation speed^68^, we doubled the masses of all hydrogen atoms and reduced non-bonded interaction cutoffs to 1 nm, which allowed us to use an integration time step of 4 fs. In the production runs, the Velocity-Rescale thermostat was used^78^.

### Preparation of the models for force spectroscopy MDS

As starting structure, we used an equilibrated frame from a multi-µs simulation of four copies of Spike proteins^68^. The initial part of the stalk was remodeled as a right-handed coiled-coil^10^ using a recent structure from Cai et al.^23^ (PDBid 6xr8) and reconnecting the flexible loops using MODELLER^79^. In addition, we cleaved each monomer at the Furin cleavage site (after R685). An equilibrated structure of the RBD bound to the ACE2 receptor was taken from Mehdipour et al.^33^. The RBD domain was aligned with the one in a full Spike model, so that residues flanking the domain (336 and 517) were in close proximity in both structures, and the ACE2-bound system was not clashing with the Spike. The original RBD (336 to 517) was then removed and the system energy minimized. The procedure was then repeated for systems with 2 and 3 ACE2 receptors bound to 2 and 3 RBDs, respectively. Both ACE2 and Spike retained a full set of glycans, as described^10,33,68^. For steric reasons, our analysis did not consider the possibility of the two peptidase domains in an ACE2 dimer being simultaneously bound to two RBDs of a single Spike. To reduce the system size and facilitate pulling simulations, we truncated Spike proteins at residue 1162. Transmembrane domains of all ACE2 receptors in a given system were embedded in circular nanodiscs of a plasma membrane, as described^33^. Nanodiscs were constructed via CHARMM-gui^80^ and stabilised with standard membrane scaffold proteins (MSP) as implemented in the nanodisc builder^81^. In addition to the truncated systems used for pulling, we set up a large simulation box containing a full-length Spike embedded in a virial membrane-bound to a nanodisc-embedded ACE2. All systems were embedded in hexagonal boxes and solvated. To mimic an extracellular ion composition, we supplemented simulated systems with 0.15 mM of NaCl. The final system sizes and numbers of atoms are shown in Supplementary Table 1.

### Pulling simulations

Constant velocity pulling simulations were performed using the GROMACS pull code. A pulling coordinate was defined between the center of mass of the terminal residues of a truncated Spike and the nanodisc, including transmembrane domains of the ACE2 receptor. The pulling force was applied only along the longest axis of the simulation box and the spring constant was kept at 1000 kJ/(mol nm^2^). For each system, we performed pulling with two velocities: 0.01 and 0.1 nm/ns, resulting in nominal loading rates of 0.0166 and 0.166 N/s, respectively. Instantaneous force values were recorded at 200 or 20 ps intervals for the slower and faster pulling, respectively.

### Analysis of the pulling simulations

To determine the optimal width for force averaging, we calculated an autocorrelation of the force fluctuations in the single ACE2 system (pulling velocity of 0.01 nm/ns), after the Spike:ACE2 bond had been severed, and before any interaction of the moving Spike protein with periodic images of ACE2. Force values became consistently un-correlated between 300 and 1000 ps. We chose a conservative 10 ns window for averaging.

To calculate the detachment forces, we monitored the fraction of native interface contacts Q for each Spike:ACE2 pair at 1 ns intervals. We determined the detachment force as the maximum of the averaged force in the 10 ns preceding the drop of Q below 30%. If two consecutive Spike:ACE2 bonds were broken within 10 ns from each other, we assumed this to be a simultaneous event and reported the averaged value of both detachment force and detachment extension instead.

### Detection of the unbinding mechanism

We selected two clusters of residues at two opposite ends of the elongated Spike:ACE2 interface: Y505 on Spike and K353 on ACE2, and F486+Y489 on Spike and T27+Y83 on ACE2. We monitored the distance between the centers of mass of these clusters every 1 ns, averaged over 10 ns blocks (except for the shortest trajectory of Spike:1ACE2 at 0.1 nm/ns, where no averaging was used). When the distance exceeded 2× the distance in the initial frame, we recorded the time as a detachment time of this part of the interface. The time difference between the events was recorded as a lag time. To determine the total opening of the interface we quantified the distance within the cluster that ruptured first at the moment of the failure of the second cluster and subtracted from it the distance in the starting configuration.

### Residue–residue contacts

Two residues (a protein amino acid or a glycan monosaccharide) are defined to be in contact with each other if at least one non-hydrogen atom pair is within 3.5 Å of each other.

### Fraction of native interface contacts

Native contacts are defined as the contacts present within the native state. Two heavy atoms *i* (from ACE2) and *j* (from Spike) are considered to form a native contact if their distance *r*_*ij*_^0^ in the initial structure is <4.5 Å. The fraction of native contacts *Q* is then defined in a configuration *X* as

5 $$Q\left({r}_{{ij}}\left(X\right),\,{r}_{{ij}}^{0}\right)=\frac{1}{N}\mathop{\sum}\limits_{i}\mathop{\sum}\limits_{j}\frac{1}{1+{e}^{\left[\beta ({r}_{{ij}}\left(X\right)-\lambda {r}_{{ij}}^{0})\right]}}$$

where the double sum runs over the *N* distinct pairs of native contacts (*i*, *j*) and *r*_*ij*_(*X*) is the distance between heavy atoms *i* and *j* in configuration *X*. We set the smoothing and padding parameters to *β* = 5 Å^−1^ and *λ* = 1.8, respectively.

### Functionalization of AFM cantilever tips with SARS-CoV-2 Spike trimer or RBD monomer

AFM cantilever tips (Bruker, MSCT) were washed in chloroform (VWR Chemicals, 22711.324) (3 × 5 min), dried, treated with ozone plasma for 15 min, washed in isopropanol (VWR Chemicals, 20842.330) (3 × 5 min), washed in Millipore water (3 × 5 min), and finally dried at 180 °C in air. The cleaned cantilevers were amino-functionalized using the gas phase method for reaction with APTES ((3-Aminopropyl)triethoxysilane) (Sigma-Aldrich, 440140). Afterward, the cantilevers were pegylated by incubation for 1 h in 0.3 mL chloroform containing 15 µL trimethylamine (Sigma-Aldrich, 90335) and 1 mg polyethylene glycol (PEG) linker with two different end groups: N-hydroxysuccinimide (NHS) and maleimide, resulting in the acylation of the surface-bound amino groups. The cantilevers were then washed with chloroform (×3) and dried. Subsequently, cantilevers were immersed for 4 h in a mixture prepared by adding the following materials in the exact order: 50 µL of 2 mM thiol-trisNTA (provided by R.W. and R.T., Institute of Biochemistry, Goethe-Universität Frankfurt), 1 µL of 100 mM EDTA (pH 7.5), 2.5 µL of 1 M HEPES (pH 7.5), 1 µL of 100 mM tris(carboxyethyl)phosphine (TCEP) hydrochloride, and 1.25 µL of 1 M HEPES (pH 9.6) buffer. Then cantilevers were washed with TRIS or HEPES buffered saline (TBS or HBS, pH 7.4). For the final protein conjugation step, cantilevers were incubated overnight at room temperature in the following solutions: (1) 186 µg/mL His_6_-tagged standard strain SARS-CoV-2 Spike trimer from HEK in HBS, (2) 235 µg/mL His_6_-tagged standard strain SARS-CoV-2 RBD monomer from HEK in TBS, (3) 200 µg/mL His_6_-tagged SARS-CoV-2 Spike mutant N234Q trimer from HEK in HBS (provided by M.K.) in TBS, (5) 50 µg/mL His_11_-tagged SARS-CoV-2 RBD monomer mutant N501Y from HEK (Sino Biological), (6) 50 µg/mL His_11_-tagged SARS-CoV-2 RBD monomer mutant E484K from HEK (Sino Biological), (7) 150 µg/mL His_10_-tagged SARS-CoV-2 Delta variant RBD monomer from HEK (AcroBiosystems), (8) 150 µg/mL His_10_-tagged SARS-CoV-2 Delta variant Spike from HEK (AcroBiosystems), (9) 150 µg/mL His_11_-tagged SARS-CoV-2 Omicron (B.1.1.529) variant RBD monomer from HEK (Sino Biological), and (10) 150 µg/mL His_10_-tagged SARS-CoV-2 Omicron (B.1.1.529) variant Spike from HEK (AcroBiosystems). All solutions contained additionally 200 µM NiCl_2_. Cantilevers were then stored at 4 °C and washed three times in measurement solution before measurements.

### Cells

VeroE6 cells were provided by J.P. (Institute of Molecular Biotechnology of the Austrian Academy of Sciences (IMBA), original source: ATCC CRL-1586). Cells were grown on plastic Petri dishes (with a diameter of 35 mm) using DMEM (Biochrom, FG0445), containing 10% FBS (Gibco, 1600-044), 500 unit/mL penicillin and 100 µg/mL streptomycin (Thermo Fisher Scientific, 15140122). For AFM measurements, the density of the cells was at about 10–30% coverage of the dish surface. 2 mL of growth medium was poured away and one or two mL of physiological HEPES (4-(2-hydroxyethyl)-1-piperazineethanesulfonic acid) buffer containing 140 mM NaCl, 5 mM KCl, 1 mM MgCl_2_, 1 mM CaCl_2_, and 10 mM HEPES (pH 7.4 with NaOH) was added. For measurement with RBD monomer, 50 µM NiCl_2_ was supplemented.

### Recombinant production of the SARS-CoV-2 Spike, RBD and Spike N234Q glycomutant

Genetic constructs. A pCAGGS vector encoding the pre-fusion stabilized ectodomain of the SARS-CoV-2 Spike protein from the Wuhan-1 isolate (NCBI Reference sequence: YP_009724390.1) with a C-terminal thrombin cleavage site, T4 foldon trimerization domain, and hexa-histidine tag and a pCAGGS vector encoding the Spike receptor binding domain (residues R319-F541) with a C-terminal hexa-histidine tag was kindly provided by Florian Krammer, Icahn School of Medicine at Mount Sinai, NY^21^. The stabilized Spike ectodomain gene sequence contained two proline mutations at positions K986P and V987P and the polybasic cleavage site (RRAR) was replaced by a single alanine residue. All sequences had been codon-optimized for expression in mammalian cells. The Spike sequence was re-cloned into a pTT28 mammalian expression vector (National Research Council, NRC, Ottawa, Canada) and the Spike glycomutant N234Q was generated by inverse PCR using phosphorylated mutagenic oligos (Integrated DNA Technologies, Leuven, BE). Following site-directed mutagenesis PCR, the parental plasmid was digested with *Dpn*I, the amplified mutant vector was gel-purified, self-ligated, and transformed into competent cells Neb5α *E. coli* cells (both New England Biolabs, Ipswich, MA). Mutated sequences were confirmed by Sanger sequencing (Microsynth, Balgach, CH).

Recombinant expression in HEK293-6E cells. Recombinant Spike RBD, trimeric wildtype Spike as well as Spike glycomutant N234Q were expressed in HEK293-6E suspension cells (licensed from National Research Council, NRC, Ottawa, Canada), as previously described^19,82^. Briefly, cells were cultivated in Freestyle™ F17 medium supplemented with 4 mM l-glutamine, 0.1% (v/v) Pluronic F-68, and 25 µg/mL G-418 (all Thermo Fisher Scientific, Waltham, MA) in a humidified atmosphere of 8% (v/v) CO_2_ at 37 °C shaking at 130 rpm. Cells were transfected with pCAGGS-RBD, pCAGGS-S, or pTT28-S-N234Q by the dropwise addition of a mixture of 1 µg plasmid DNA and 2 µg linear 25-kDa polyethyleneimine (PEI) (Polysciences, Inc., Hirschberg, DE) per mL of culture volume (~2.0 × 10^6^ cells/mL, 1000 mL total). 2- and 4 days post-transfection, cells were supplemented with 0.5% (w/v) tryptone N1 (Organotechnie, La Courneuve, FR) and 0.25% (w/v) d(+)-glucose (Carl Roth, Karlsruhe, DE). Expression supernatants were harvested five days post-transfection, were clarified (1000×*g*, 10 min, 4 °C; 10,000×*g*, 30 min, 4 °C) and 0.45 µm-filtrated before subsequent downstream procedures.

### Purification of the Spike proteins

The recombinant wildtype Spike trimer and RBD monomer were purified essentially as in refs. 14,19,82. Expression supernatants containing his-tagged Spike N234Q were supplemented with 0.05% (v/v) Tween-20 (Fisher Scientific, Hampton, NH), were about 7-fold concentrated and diafiltrated against 20 mM sodium phosphate buffer supplemented with 500 mM NaCl, 0.05% (v/v) Tween-20 and 20 mM imidazole (pH 7.4) using an Äkta Flux System (Cytiva, Marlborough, MA) equipped with a Pellicon XL Biomax 50 kDa, 0.1 m^2^ ultrafiltration module (Merck, Darmstadt, DE). The protein was captured on a 5-mL HisTrap FF Crude immobilized metal affinity chromatography (IMAC) column on an Äkta Pure system (all from Cytiva, Marlborough, MA) equilibrated with 20 mM sodium phosphate buffer supplemented with 500 mM NaCl, 20 mM imidazole and 0.05% (v/v) Tween-20, pH 7.4. Following two wash steps with 10% and 20% elution buffer (20 mM sodium phosphate buffer supplemented with 500 mM NaCl, 500 mM imidazole, and 0.05% (v/v) Tween-20 (pH 7.4), the protein was eluted by applying a linear gradient from 20 to 500 mM imidazole over 5 column volumes. Fractions containing Spike N234Q were concentrated and diafiltrated to PBS + 0.05% (v/v) Tween-20 using Amicon Ultra Centrifugal Filter Units with a 100 kDa cut-off (Merck, Darmstadt, DE). Purity was assessed by SDS PAGE. Protein concentration was determined by measuring the absorbance at A280 on a NanoDrop^TM^ 1000 spectrophotometer (Thermo Fisher Scientific, Waltham, MA) using extinction coefficients estimated from the respective amino acid sequences with the Expasy ProtParam tool. Proteins were stored at −80 °C until further use.

### Preparation of soluble recombinant human ACE2

Clinical-grade soluble recombinant human ACE2 (amino acids 1–740) was produced by Polymun Scientific (contract manufacturer) from CHO cells according to Good Manufacturing Practice guidelines and formulated as a physiologic aqueous solution.

### SMFS measurements

All force–distance curves were recorded at room temperature using a PicoPlus 5500 AFM setup with the software PicoScan 5 (Agilent Technologies, Chandler, AZ, USA) on living cells with the assistance of a CCD camera for localization of the cantilever tip on selected cells. The optical system of the AFM was focused on the cantilever tip, while the sample plate with the Petri dish was moved upwards by the step motor. Before the cells on the dish reached the focus, the piezo tube of the AFM started to scan in the *z*-axis with a scanning range of 3 µm and at a scanning frequency of 1 Hz. Then, the sample plate was moved upwards by the step motor using manual control with 1 µm per step. Due to the resistance of the liquid, a gap between the approaching curve and the retraction curve appeared, when the AFM tip was close to the sample surface. About 2 µm before the AFM tip touched the sample surface, the approaching curve was no longer parallel to the retraction curve. With this signal, the movement of the step motor was stopped. A further approach was accomplished by gradually changing the voltage on the piezo tube. With this approach method, the indentation force is kept below 30 pN in the initial contact between the AFM tip and the sample surface.

The functionalized cantilever with a nominal spring constant of 0.01 N/m was moved downwards to the cell surface and moved upwards after the deflection of the cantilever reached the force limit. The deflection (*z*) of the cantilever was monitored by a laser beam on the cantilever surface and plotted versus the *z*-position of the scanner, from which the force (*F*) can be determined according to Hooke’s law (*F* = *kz*, with *k* being the cantilever spring constant). When the tip-tethered molecule bound to ACE2 on the cell surface, a pulling force developed during the upward movement of the cantilever causing the cantilever to bend downwards. At a critical force, i.e. the unbinding force, the tip-tethered molecule detached from ACE2, and the cantilever jumped back to its neutral position. Dynamic force spectroscopy measurements were performed by varying the force loading rate which is the product of the pulling velocity multiplied by the effective spring constant. For this, the sweep range was fixed at 3000 nm, but the sweep rate varied from 0.25 to 2 Hz. For each cellular position and sweep rate, 100–200 force–distance cycles with 2000 data points per cycle and a typical force limit of about 30 pN were performed. The spring constants of the cantilevers were determined using the thermal noise method.

### Force data analysis

For the force data analysis, Matlab R2013a and OriginPro 9.0 were used. The unbinding event was identified by local maximum analysis using a signal-to-noise threshold of 2. The binding activity was calculated as the fraction of curves showing unbinding events. For example, if 150 curves from 1000 measured curves show unbinding events, the binding activity is 15%. Two-tailed Student’s *t*-test was performed for statistical analysis. The unbinding force and effective spring constant (slope before rupture) were determined from force curves showing unbinding events. The force loading rate (*r*) of every individual curve was calculated by multiplying the effective spring constant with the pulling speed.

The probability density function (PDF) of the unbinding force was constructed from every unbinding event on the same cell at the same pulling speed. For each unbinding force value, a Gaussian of the unitary area with its center representing the unbinding force and the width (standard deviation) reflecting its measuring uncertainty (square root of the variance of the noise in the force curve) was computed. All Gaussians from one experimental setting were accordingly summed up and normalized with their binding activity to yield the experimental PDF of unbinding force. The maximum of the PDF reflects the most probable unbinding force of the bond and can be easily extracted with Gaussian fitting according to a standard procedure in the literature. Likewise, two or three maxima in the force PDF were extracted by fitting with a multi-Gaussian function, from which the most probable unbinding forces of two or three bonds were determined.

Unbinding events within the mean force ± standard deviation of the Gaussian fit of the first peak of the force PDF were used to create an unbinding force vs. force loading rate plot, to show dynamic aspects of the bond. The Bell–Evans theory^30^ was employed to fit the width of the energy barrier *x*_B_ and the dissociation rate constant *k*_off_ for the obtained data.

The force of multiple bonds was calculated from William’s Markov binding model^45^, which assumes uncorrelated bond dissociation without mechanical coupling between individual bonds. Using the parameters obtained from fitting the single bonds with the Bell–Evans model, we obtained

6 $${r}_{{\rm {f}}}={k}_{{{{{{\rm{off}}}}}}}\frac{{k}_{{{{{{\rm{B}}}}}}}T}{{x}_{\beta }}{\left[\mathop{\sum }\limits_{l=1}^{{N}_{{{{{{\rm{B}}}}}}}}\frac{1}{{l}^{2}}\exp \left(-\frac{{F}^{*}{x}_{\beta }}{l{k}_{{\rm {B}}}T}\right)\right]}^{-1}$$

where *r*_f_ is the force loading rate, *k*_B_ is the Boltzmann constant, *T* is the temperature, F* is the unbinding force, and *N*_B_ is the number of bonds.

To acquire the kinetic on-rate *k*_on_, the measured data of binding activity *P* versus contact time *t*_c_ were fitted with the following function^26^, representing pseudo-first-order kinetics: *P* = *A*(1−exp(−(*t*_c_−*t*_0_)/*τ*)), where *t*_0_ is the lag time, *A* is the maximum observable binding activity, and *τ* is the interaction time. The kinetic on rate *k*_on_ is obtained as: *k*_on_ = 1/(*τC*_eff_), where *C*_eff_ is the effective concentration of the conjugated molecule on the cantilever tip. *C*_eff_ is determined as *C*_eff_ = 1/(*A*_c_*V*), where *A*_c_ is the Avogadro constant, and *V* is the volume of the hemisphere with the radius equal to the length of the crosslinker plus the length of the conjugated molecule. For the second bond or the third bond, the on rate is equal to 1/*τ*.

Errors in the kinetic rates and *K*_D_ values rates were put in the exponent as logarithmic errors, as our fits contain normal errors on log(*k*), but not on *k*.

### Modeling of ACE2:Spike bound lifetimes and avidity gains

Shear forces (*f*) exerted by moving air, mucus, or blood stress the attachment of a virus to a host cell. Forced breaking of Spike:ACE2 interactions and the resulting detachment compete with cell internalization, which is expected to occur on a minutes time scale^83^. We used the measured kinetics of Spike:ACE2 binding under force to model the lifetimes of Spike:ACE2 complexes and the gains in lifetime resulting from multiple RBD:ACE2 interactions.

For the force-dependent rates of a single bound RBD, we used the Bell model^44^, and for the simultaneous detachment of two and three RBD:ACE2 bonds loaded in parallel the Williams model^45^

7 $${k}_{{{{{{\rm{off}}}}}}}\left(f\right)=\,{k}_{{{{{{\rm{off}}}}}}}\left(0\right)\,\exp \left(\frac{{x}_{\beta }f}{{k}_{{{{{{\rm{B}}}}}}}T}\right)$$

8 $${k}_{{{{{{\rm{off}}}}}}2}\left(f\right)=\,\frac{1}{\frac{1}{{k}_{{{{{{\rm{off}}}}}}}\left(f\right)}+\frac{1}{2{k}_{{{{{{\rm{off}}}}}}}\left(f/2\right)}}$$

9 $${k}_{{{{{{\rm{off}}}}}}3}\left(f\right)=\,\frac{1}{\frac{1}{{k}_{{{{{{\rm{off}}}}}}}\left(f\right)}+\frac{1}{2{k}_{{{{{{\rm{off}}}}}}}\left(f/2\right)}+\frac{1}{3{k}_{{{{{{\rm{off}}}}}}}\left(f/3\right)}}$$

### Surface plasmon resonance

Spontaneous thermodynamic association and dissociation were measured with surface plasmon resonance (SPR). The measurement software is Biacore X Control Software and the analysis software is BIAevaluation 3.2 RC1. ACE2 was injected at different concentrations into chambers containing isolated SARS-CoV-2 Spike protein of the Wuhan reference strain. The experimental binding curves recorded with SPR were fitted with the “1:1 Langmuir Binding model”, and *k*_on_, *k*_off_, *K*_A_, and *K*_D_ were determined.

### Reporting summary

Further information on research design is available in the Nature Portfolio Reporting Summary linked to this article.

## Supplementary information

Supplementary information

Supplementary Movie 1.

Supplementary Movie 2.

Supplementary Movie 3.

Supplementary Movie 4.

Supplementary Movie 5.

Supplementary Movie 6.

Supplementary Movie 7.

Supplementary Movie 8.

Supplementary Movie 9.

Supplementary Movie 10.

Supplementary Movie 11.

Supplementary Movie 12.

Supplementary Movie 13.

Supplementary Movie 14.

Supplementary Movie 15.

Supplementary Movie 16.

Supplementary Movie 17.

Supplementary Movie 18.

Supplementary Movie 19.

Reporting Summary

## Data Availability

Source data are provided with this paper. The data generated in Supplementary Fig. 8 have been deposited in the database Zenodo under the accession code 10.5281/zenodo.7401939.

## Code availability

Code for simulation of high speed AFM imaging is available at: https://github.com/matsikora/AFM_emulator^84^.
