# Supplementary material for: Force-tuned avidity of spike variant-ACE2 interactions viewed on the single-molecule level
Source: Nat Commun. 2022 Dec 24;13:7926. doi: 10.1038/s41467-022-35641-3 (PMC9789309; doi:10.1038/s41467-022-35641-3)
Supplement: Supplementary file 1 — Supplementary information [file 41467_2022_35641_MOESM1_ESM.pdf]

Supplementary Information

Force-tuned Avidity of Spike Variant-ACE2 Interactions viewed on the Single-Molecule Level

Supplementary Figures 1-17

Supplementary Table 1

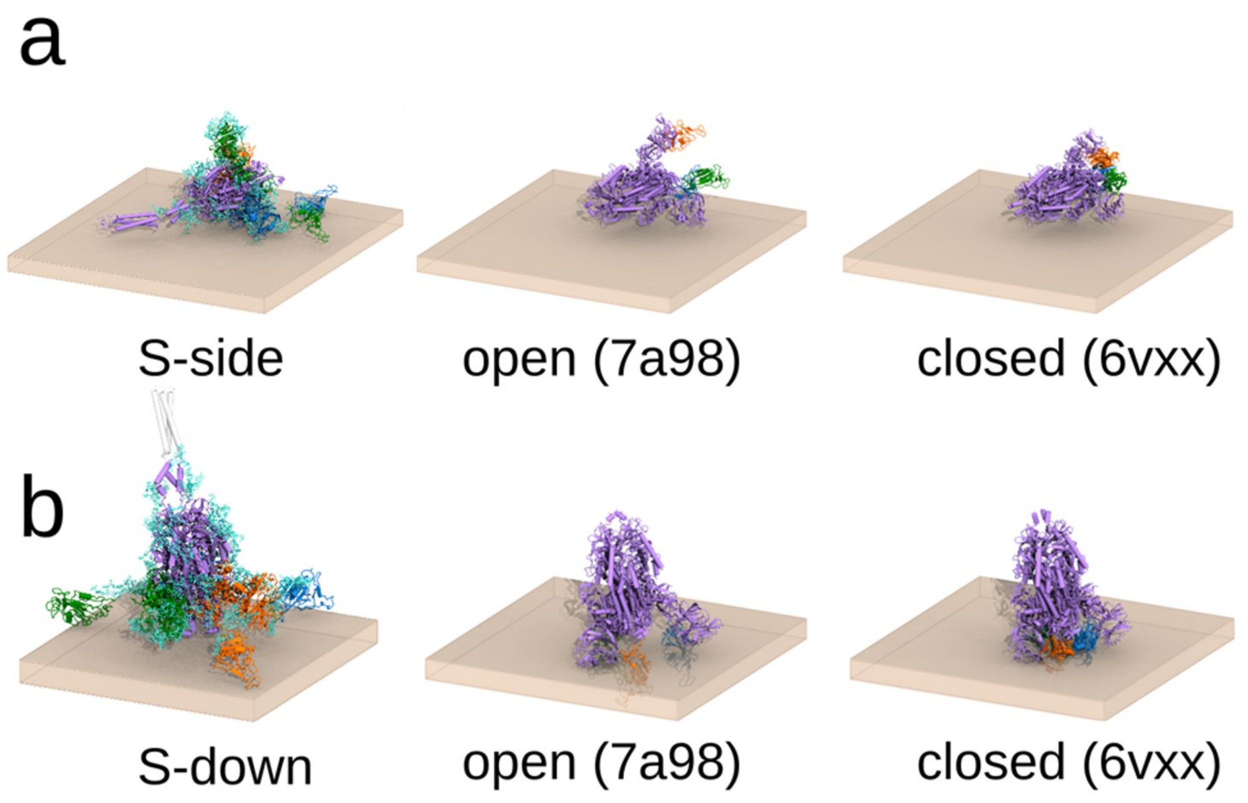

Supplementary Fig. 1.

Spike on mica surface (brown) in (a) sideways and (b) downwards orientation. (Left) Simulation model. (Center) All-RBD-up conformation (PDBid 7a98). (Right) All-RBD-down conformation (PDBid 6vxx).

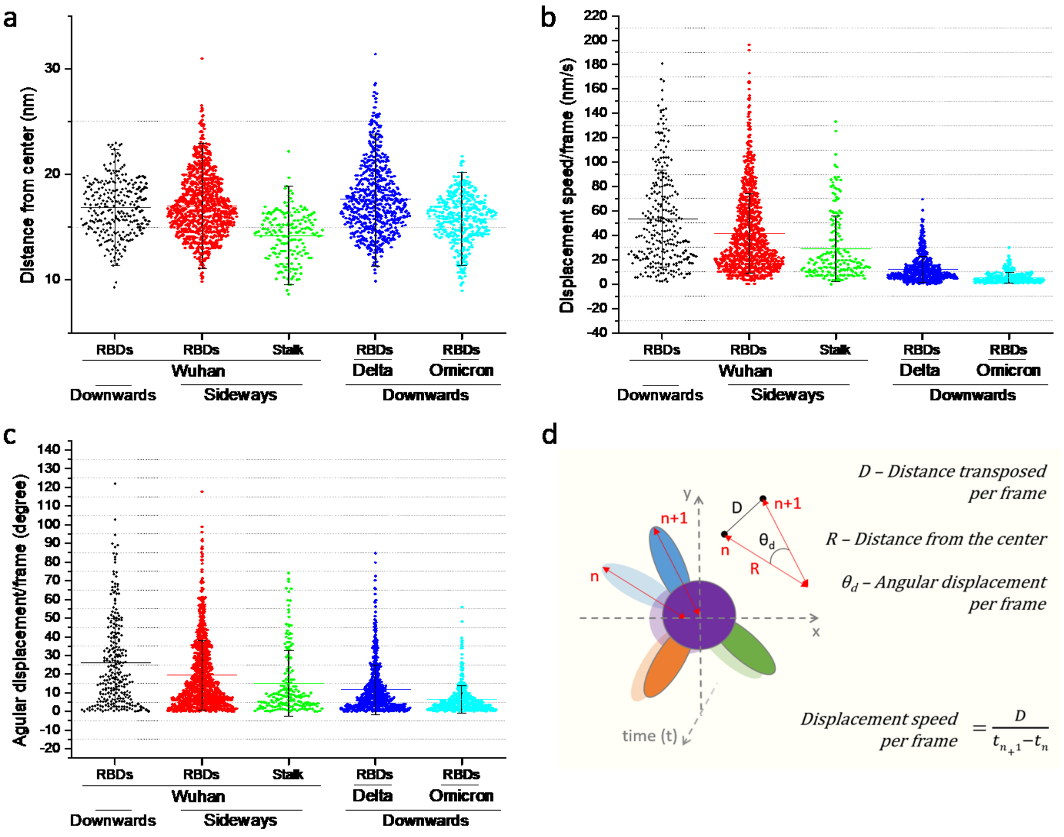

Supplementary Fig. 2.
Distance from the center, speed and angular displacement of RBDs and stalk domains. (a) Distance from the center, (b) Displacement speeds and (c) Angular displacements determined from consecutive frames of the movies. The measurements of RBDs of the Wuhan Spike in the downwards configuration are plotted as black dot, RBDs and stalk of the Spike in the sideways configuration are in red and green, respectively, and the RBDs of Delta and Omicron (B.1.1.529) Spike variants in downwards configuration are in blue and light blue, respectively. The solid horizontal lines indicate mean values and the vertical error bars standard deviation (N = 630 frames for RBDs of Wuhan Spike in downwards configuration; N = 410 frames for RBDs and stalk of Wuhan Spike in sideways configuration; N = 206 frames for Delta RBDs; N = 146 frames for Omicron RBDs). (d) Schematic of Spike protein dynamics analysis. The Spike core is represented by the purple circle and the RBDs by the blue, green and orange ellipses. The faded Spike structure represents its conformation in an initial time frame (n) and the solid structure is the evolution of its conformation in a subsequent frame (n+1). Arrows indicate the corresponding RBD positions. The positions of the Spike structures are described using cartesian coordinates in axis x and y. The axis time (t) represents the time course of a HS-AFM movie and the triangle depicts the calculations of the parameters. Source data are provided as a Source Data file.

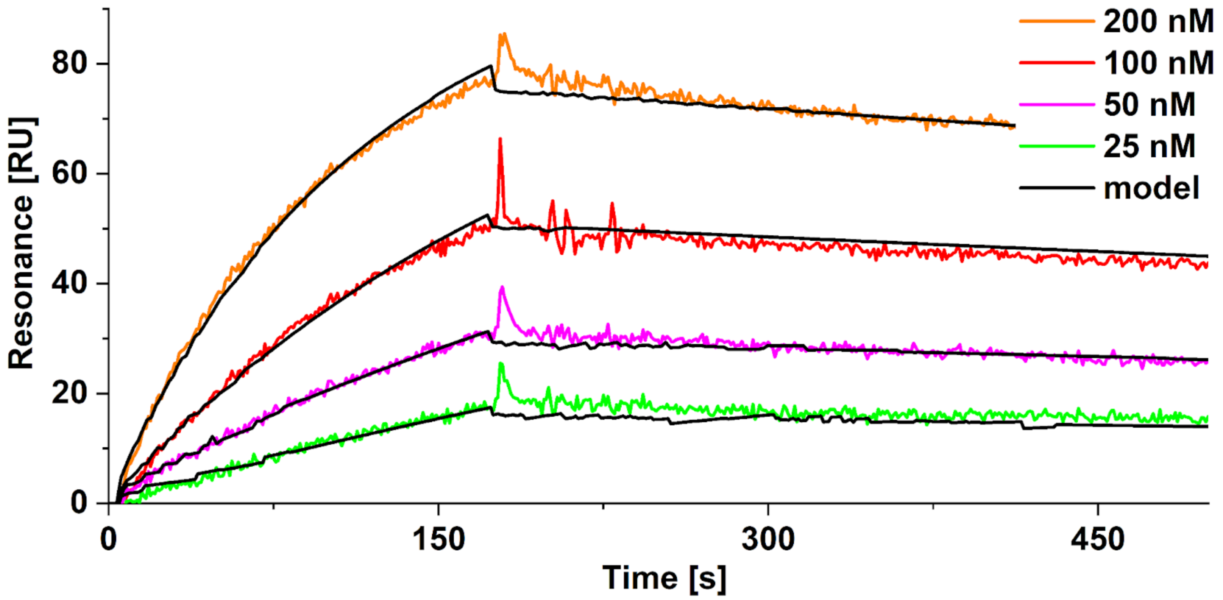

Supplementary Fig. 3.

Measurements of association and dissociation kinetics of ACE2 to Spike trimer measured by SPR. Fits yielded: $k_{on} = (3.70 \pm 0.87)x10^4\ M^{-1}s^{-1}$, $k_{off} = (2.32 \pm 1.17)x10^{-4}\ s^{-1}$. Source data are provided as a Source Data file.

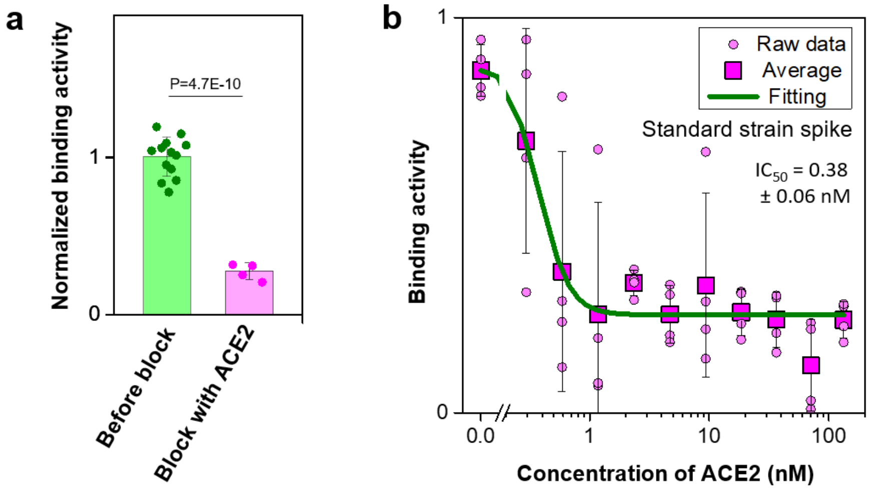

Supplementary Fig. 4.

Soluble ACE2 blocks the binding between standard strain Spike trimer on the AFM tip and ACE2 on the surface of VeroE6 cells. (**a**) Histogram of the blocking experiment. (n= 13 cells before block and 4 cells after block. The error bars indicate SD. Two-sided Welch's t-Test). (**b**) Concentration-dependent blocking of reference strain Spike trimer with soluble ACE2. (n= 4 cells. The error bars indicate SD). Source data are provided as a Source Data file.

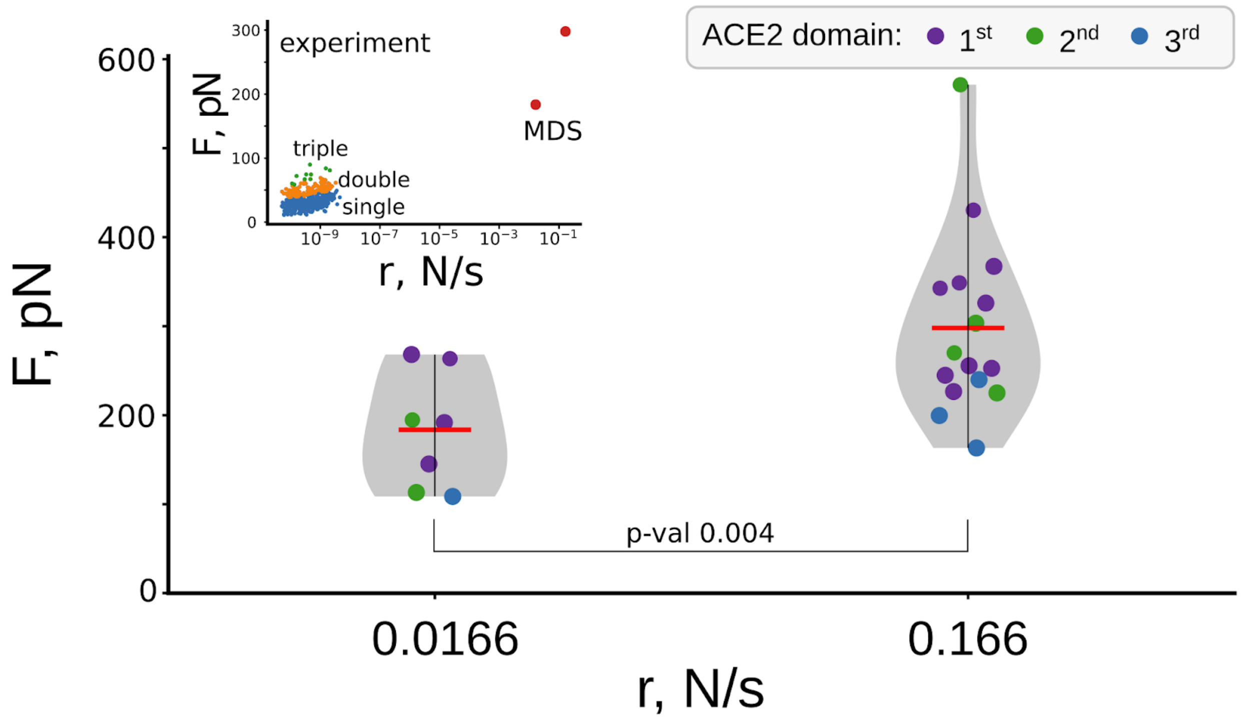

Supplementary Fig. 5.

Dependence of the distribution of the detachment forces on the loading rate (r) with individual events shown. Colors denote order of detachment of ACE2 domains. P-value was calculated using two-tailed Welch's t-test. Inset shows the average F values in the context of the SMFS measurements (cf. Fig. 2f). Source data are provided as a Source Data file.

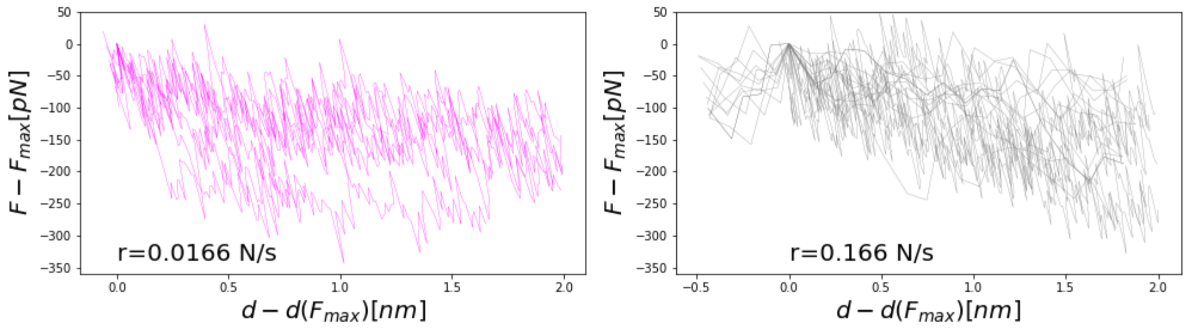

Supplementary Figure 6.

Overlay of force (F)-extension (d) traces from MD simulations with slow (left) and fast (right) pulling. Shown are traces for all detachment events of all Spike:ACE2 bonds. The traces start 5 ns before the estimated detachment point, and are shifted horizontally with the extension at Fmax set to zero and vertically by subtracting the force Fmax at the point of detachment. Source data are provided as a Source Data file.

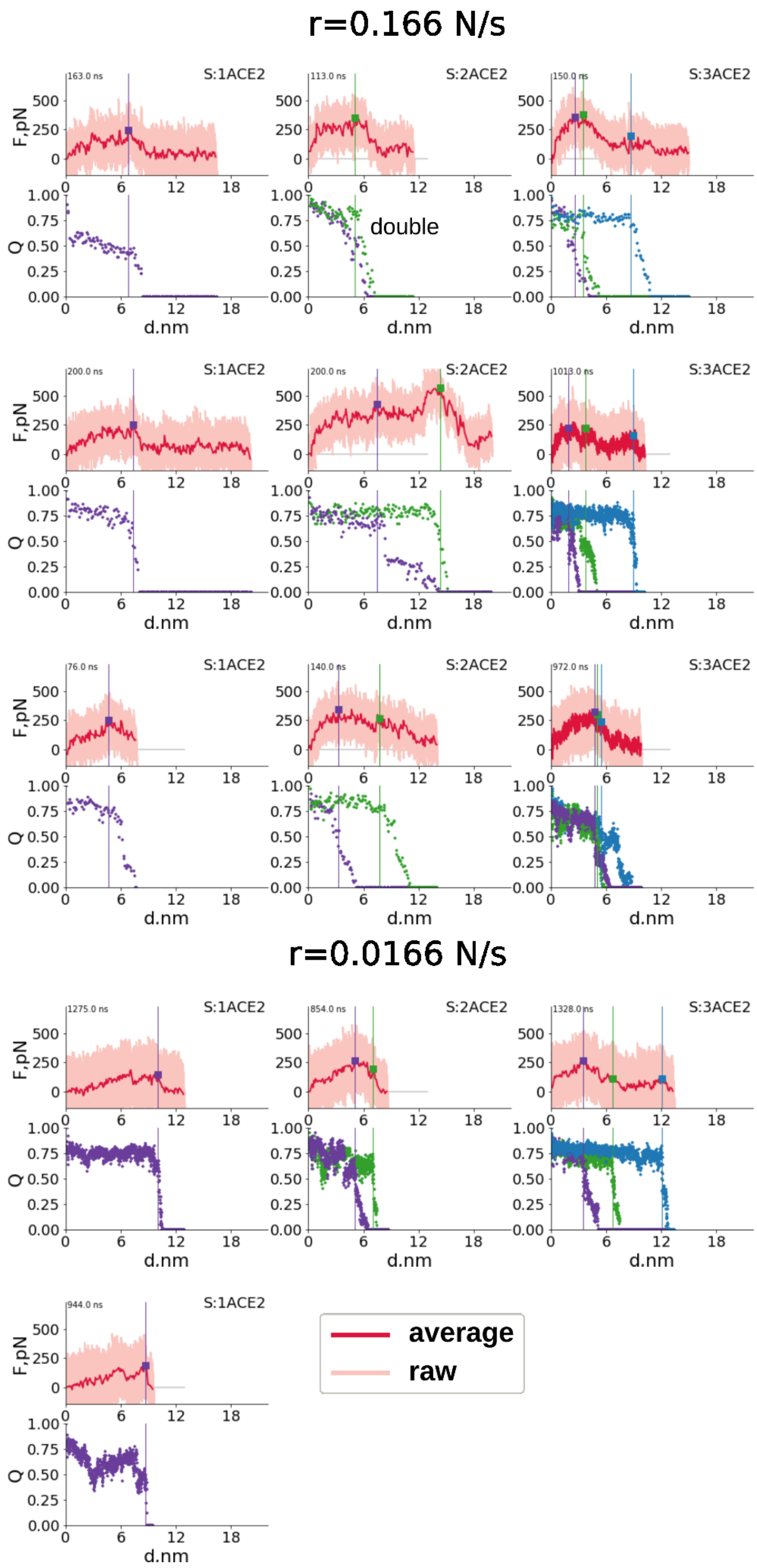
r=0.166 N/s
163.0 ns
S:1ACE2
113.0 ns
S:2ACE2
double
150.0 ns
S:3ACE2
200.0 ns
S:1ACE2
200.0 ns
S:2ACE2
1013.0 ns
S:3ACE2
76.0 ns
S:1ACE2
140.0 ns
S:2ACE2
972.0 ns
S:3ACE2
r=0.0166 N/s
1275.0 ns
S:1ACE2
854.0 ns
S:2ACE2
1328.0 ns
S:3ACE2
944.0 ns
S:1ACE2
F,pN
Q
d.nm
average
raw

Supplementary Fig. 7.

Force-extension curves and corresponding fraction of native interface contacts for all simulated systems. Simultaneous unbinding of the two ACE2s is indicated as “double”. Red traces denote window averaged values, pink lines denote raw data. Source data are provided as a Source Data file.

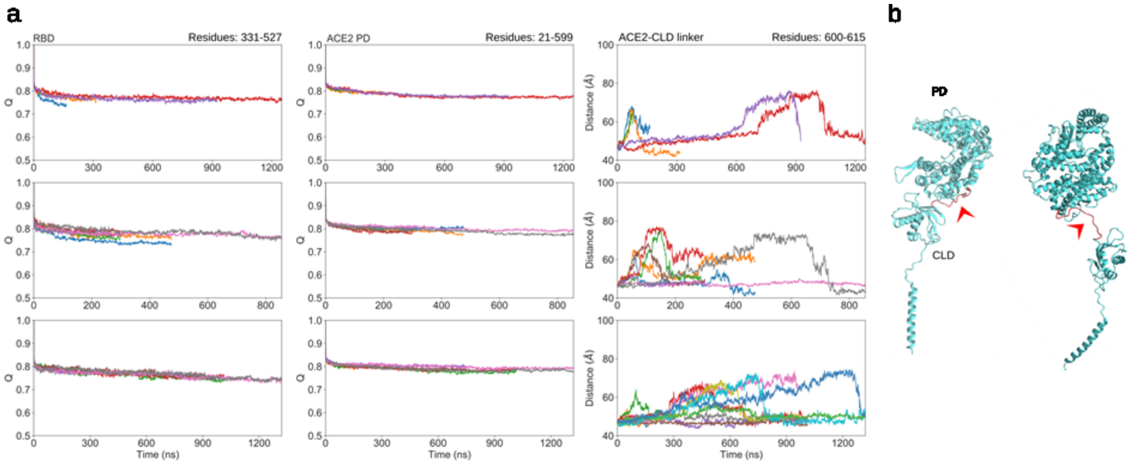

Supplementary Fig. 8.

Stability of the domains. **a)** Fraction of the native contacts Q between the RBD (left) and the ACE2 protease domain (PD, center), and extension of the linker between PD and collectrin-like domain (CLD) over time (right). Spike:1ACE2, Spike:2ACE2 and Spike:3ACE2 are shown in the top, middle and bottom row, respectively. Colors indicate different replicates within each system. b) Cartoon representation of a monomer of ACE2 with PD and CLD marked. The red section indicated with an arrowhead corresponds to the PD-CLD linker, which gets temporarily unfolded during pulling. Left and right cartoon renders show ACE2 before and after the unfolding, respectively. Source data are provided as a Source Data file.

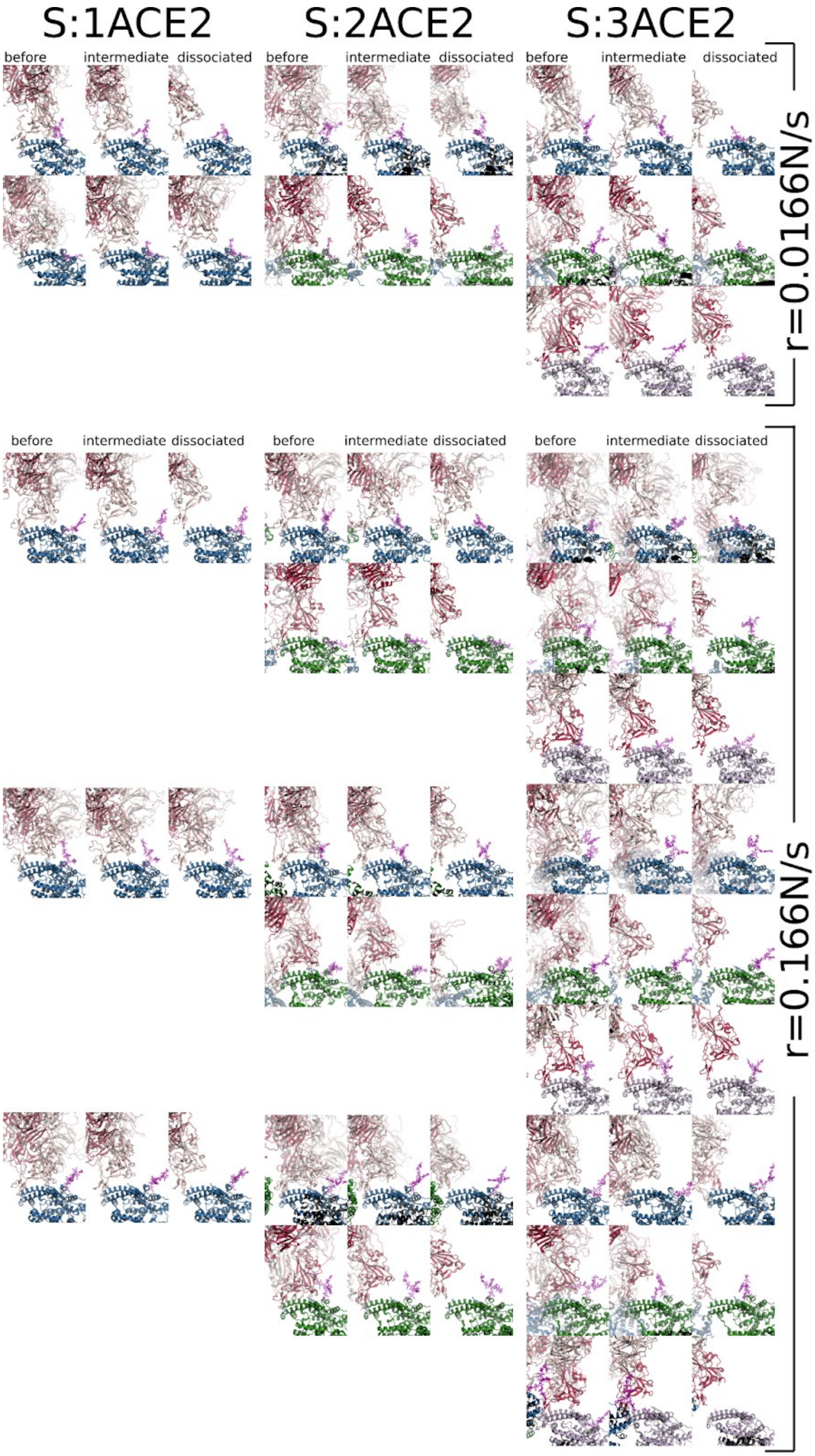
S:1ACE2
S:2ACE2
S:3ACE2
before
intermediate
dissociated
r=0.0166N/s
r=0.166N/s

Supplementary Fig. 9.

Gallery of snapshots showing the progression of the dissociation before, at the intermediate step and post dissociation for all studied systems. N322 glycan is shown as magenta sticks, Spike is on the top of each snapshot colored in shades of red, consecutive ACE2 are shown at the bottom of each snapshot in blue, green and violet.

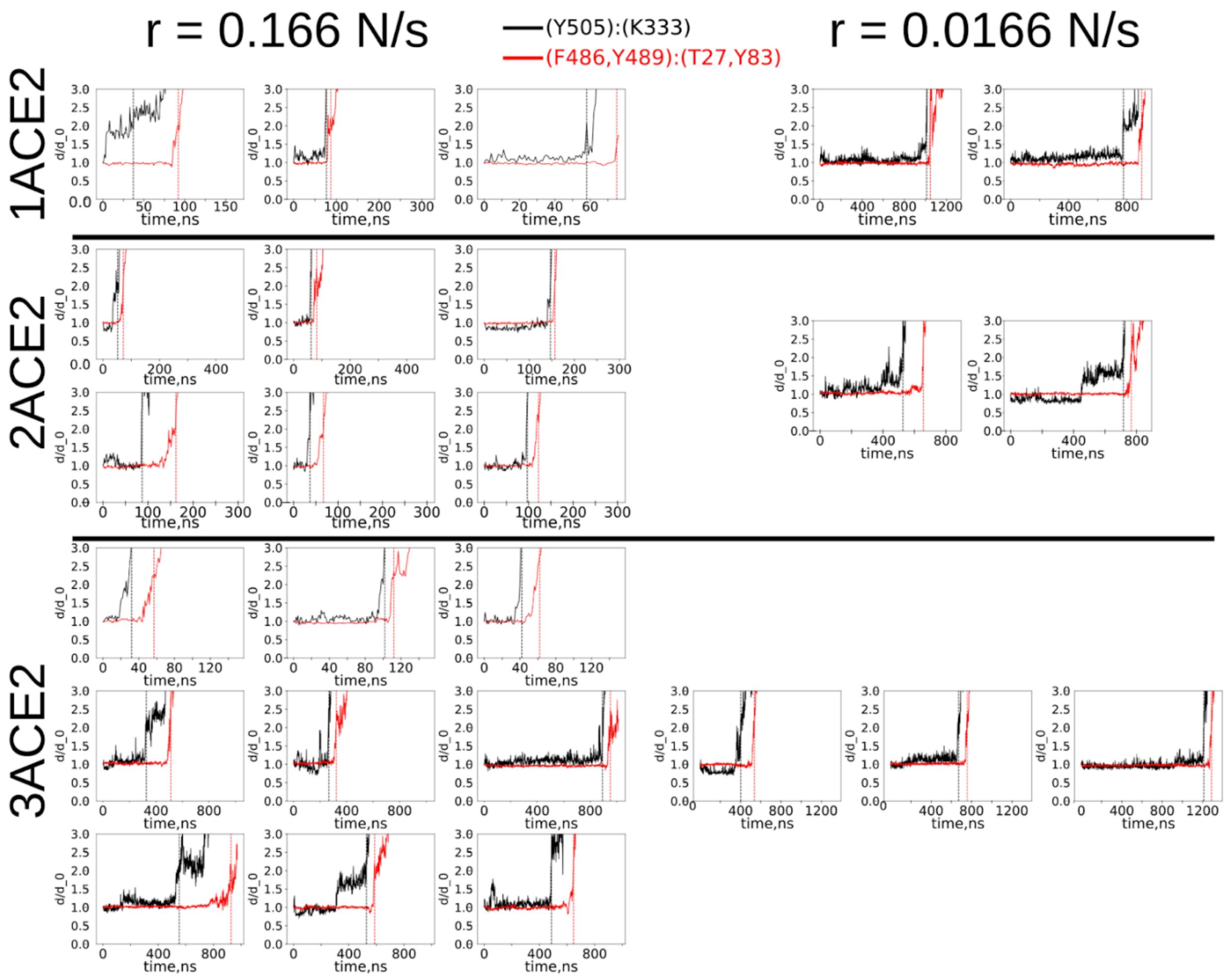

Supplementary Fig. 10.

Normalised distances (d/d_0 where d_0 is the distance at the beginning of the simulation) between centers of masses of flanking clusters of residues in each of the Spike:ACE2 interfaces and each simulation. Distances within the $F486^{S}$,$Y489^{S}$:$T27^{ACE2}$,$Y83^{ACE2}$ cluster are shown in red, $Y505^{S}$:$K353^{ACE2}$ in black, respectively. Source data are provided as a Source Data file.

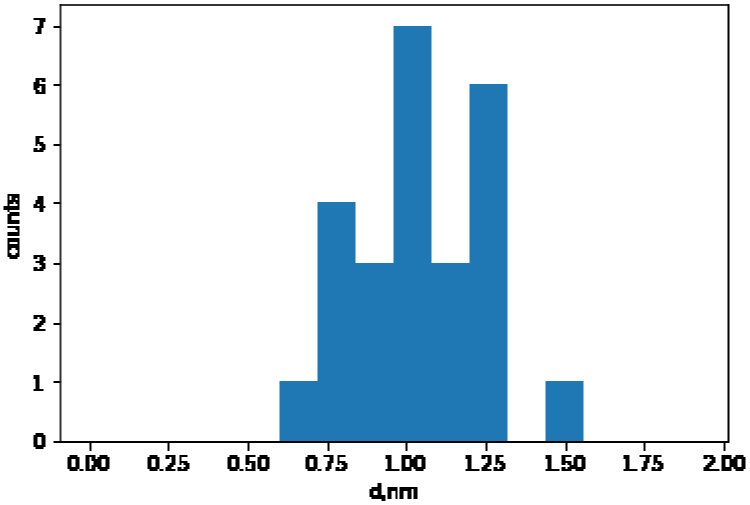

Supplementary Fig. 11.

Distribution of the opening distances for the flanking cluster $Y505^{S}$:$K353^{ACE2}$ prior to the full detachment of the complex. Source data are provided as a Source Data file.

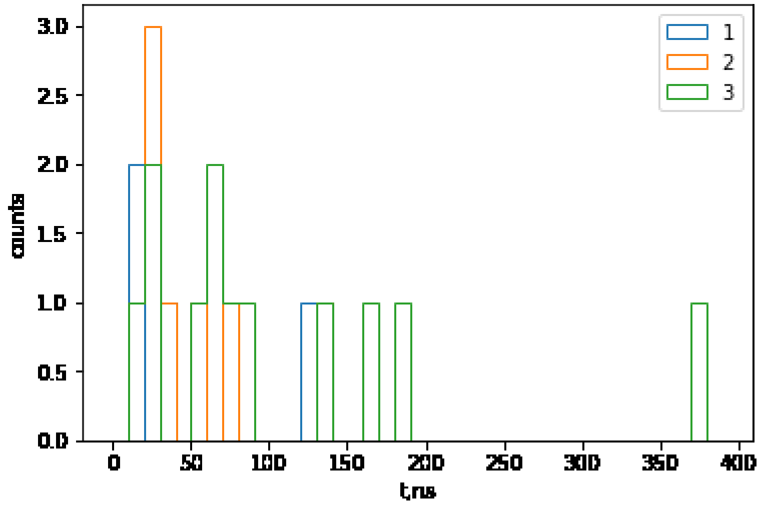

Supplementary Fig. 12.

Distribution of the lag times between the opening of the flanking cluster Y505^{S}:K353^{ACE2} and F486^{S},Y489^{S}:T27^{ACE2},Y83^{ACE2}. Color-coded are the lag times for different stoichiometry of the Spike:ACE2. Source data are provided as a Source Data file.

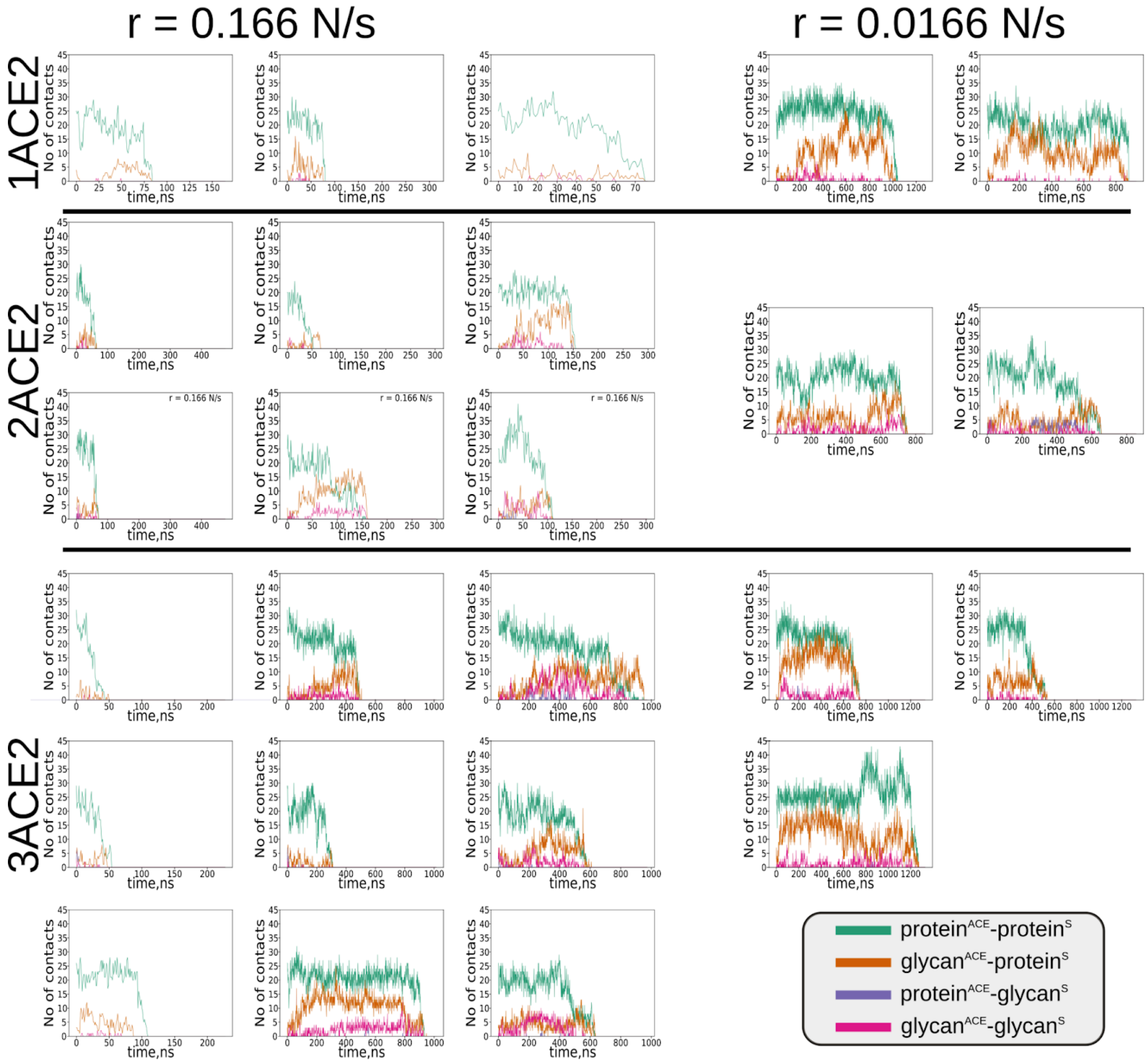

Supplementary Fig. 13.

Protein-protein, protein-glycan and glycan-glycan interactions between Spike and ACE2 over time. The panels correspond to molecular renders before, during and after the rupture as seen in Supplementary Figure 7. For detailed description of the interface please see Figure 3d. Source data are provided as a Source Data file.

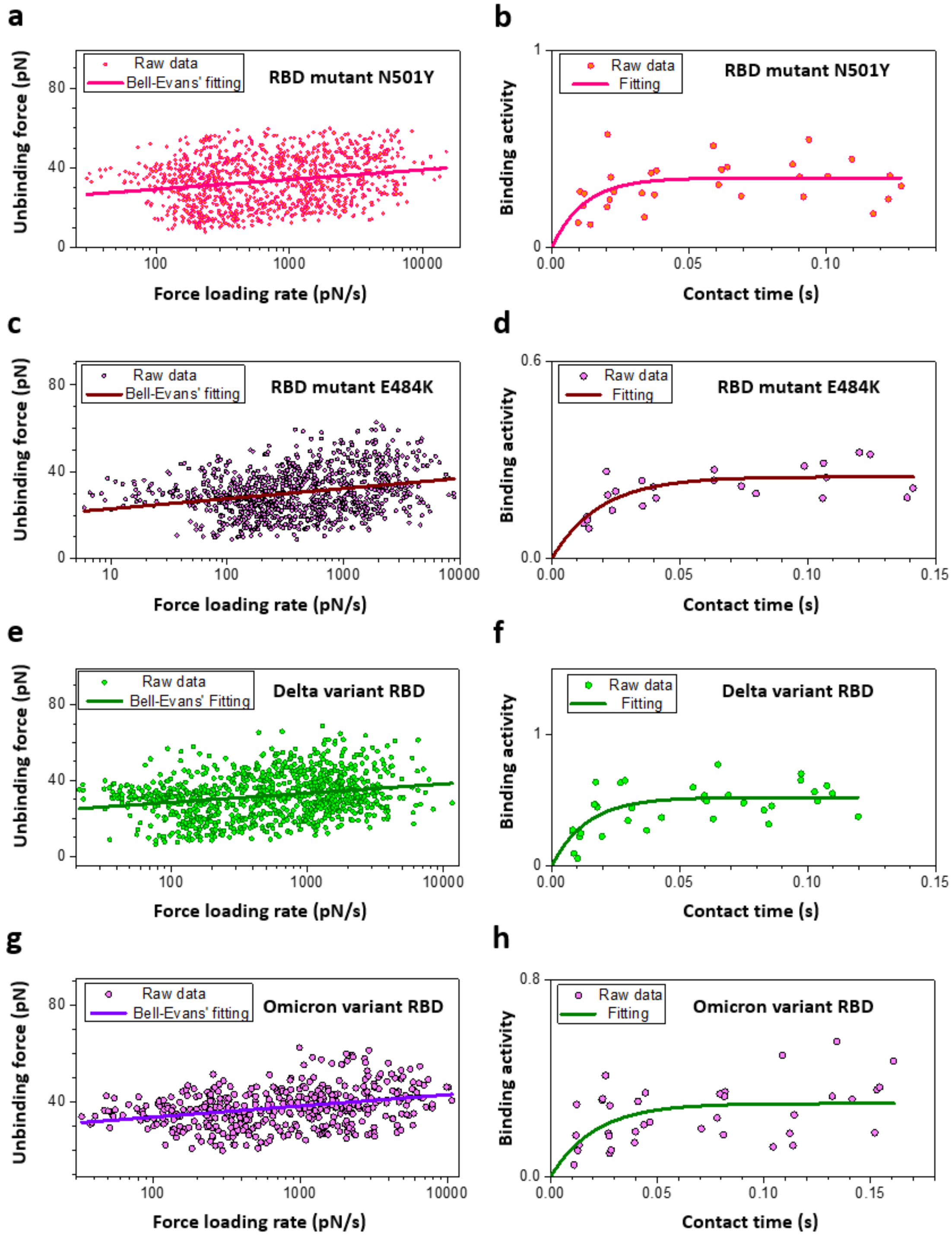

Supplementary Fig. 14.

(**a**) Unbinding force versus force loading rate and (**b**) binding activity versus contact time for RBD N501Y. (**c**) Unbinding force versus force loading rate and (**d**) binding activity versus contact time from RBD E484K. (**e**) Unbinding force versus force loading rate and (**f**) binding activity versus contact time for Delta variant RBD. (**g**) Unbinding force versus force loading rate and (**h**) binding activity versus contact time for Omicron (B.1.1.529) variant RBD. Source data are provided as a Source Data file.

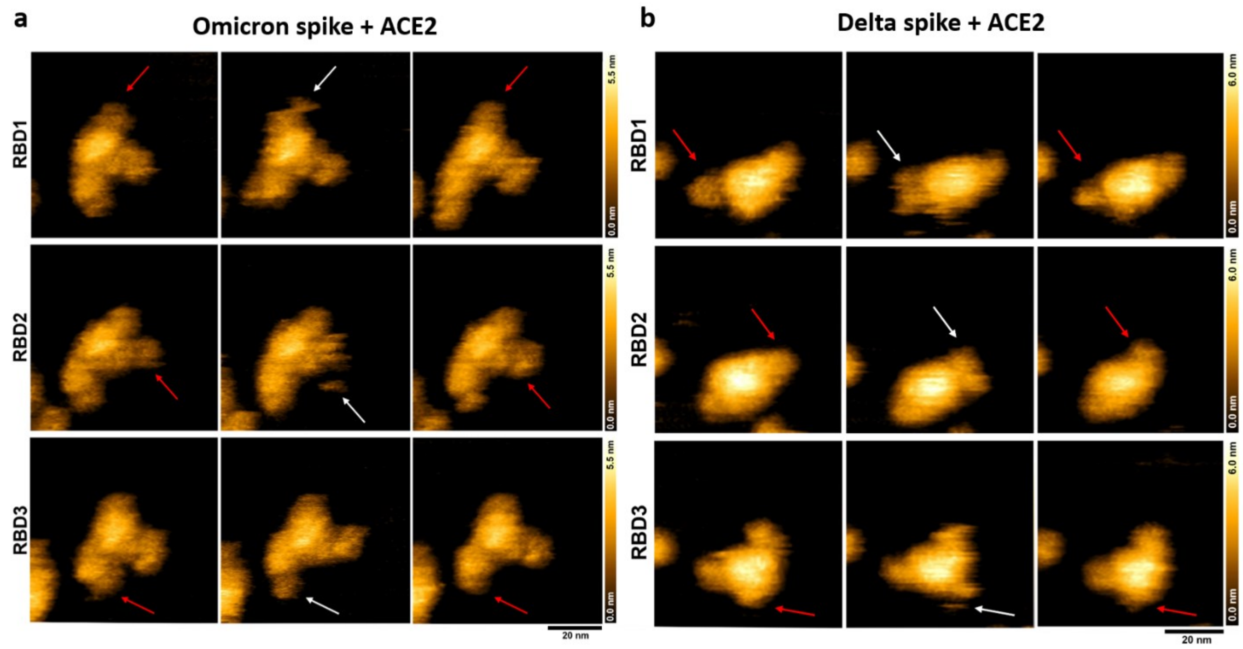

Supplementary Fig. 15.
high-speed AFM images of Omicron (B.1.1.529) (**a**) and Delta (**b**) Spikes with ACE2.

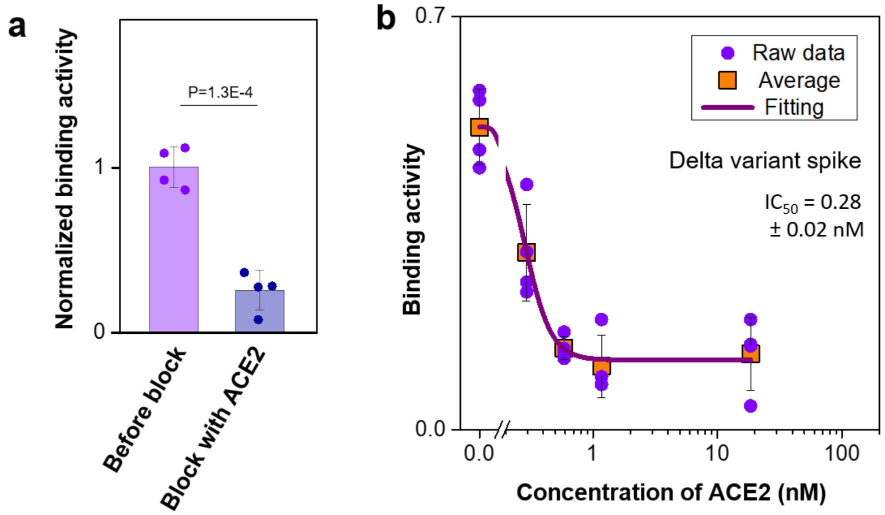

Supplementary Fig. 16.

Soluble ACE2 blocks the binding between "Delta" variant Spike trimer on the AFM tip and ACE2 on the surface of VeroE6 cells. (**a**) Histogram of the blocking experiment. (n= 4 cells. The error bars indicate SD. Two-sided Welch's t-Test). (**b**) Concentration-dependent blocking of "Delta" variant Spike trimer with soluble ACE2. (n= 4 cells. The error bars indicate SD). Source data are provided as a Source Data file.

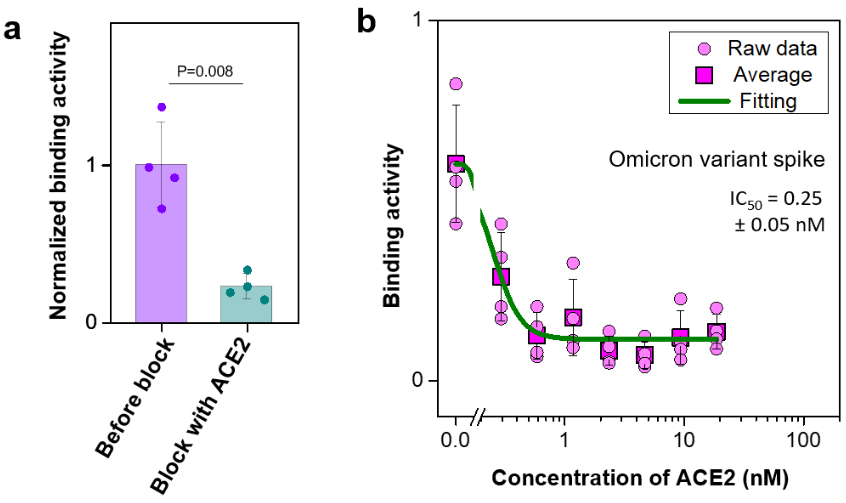

Supplementary Fig. 17.

Soluble ACE2 blocks the binding between “Omicron” variant Spike trimer on the AFM tip and ACE2 on the surface of VeroE6 cells. (a) Histogram of the blocking experiment. (n= 4 cells. The error bars indicate SD. Two-sided Welch’s t-Test). (b) Concentration-dependent blocking of “Omicron” variant Spike trimer with soluble ACE2. (n= 4 cells. The error bars indicate SD). Source data are provided as a Source Data file.

system	box size (x-y-z) [nm]	Number of atoms	Number of runs	aggregate time [µs]
Spike + 1x ACE2, full	33.44834 28.96711 57.95573	5,665,217	1 (1/0/0)	1.008
Spike + 1x ACE2	28.05757 24.29856 46.74417	3,203,907	5 (0/2/3)	2.775
Spike + 2x ACE2	28.30864 21.23141 48.40873	2,936,398	4 (0/1/3)	1.931
Spike + 3x ACE2	28.32733 21.24544 48.30436	2,936,588	4 (0/1/3)	3.814
Total time				9.528

Supplementary Table 1.

Box size, number of atomos, number of runs and aggregate times of simulated force spectroscopy systems. In parentheses, the number of runs is broken down by equilibrium simulations, and pulling at 0.01 nm/ns and 0.1 nm/ns, respectively.
